# Temporal alignment of anticipatory motor cortical beta lateralisation in hidden visual‐motor sequences

**DOI:** 10.1111/ejn.13700

**Published:** 2017-11-06

**Authors:** Simone G. Heideman, Freek van Ede, Anna C. Nobre

**Affiliations:** ^1^ Oxford Centre for Human Brain Activity Wellcome Centre for Integrative Neuroimaging Department of Psychiatry University of Oxford Oxford UK; ^2^ Brain and Cognition Lab Department of Experimental Psychology University of Oxford Oxford UK

**Keywords:** beta oscillations, magnetoencephalography, sequence learning, serial reaction time task, temporal orienting

## Abstract

Performance improves when participants respond to events that are structured in repeating sequences, suggesting that learning can lead to proactive anticipatory preparation. Whereas most sequence‐learning studies have emphasised spatial structure, most sequences also contain a prominent temporal structure. We used MEG to investigate spatial and temporal anticipatory neural dynamics in a modified serial reaction time (SRT) task. Performance and brain activity were compared between blocks with learned spatial‐temporal sequences and blocks with new sequences. After confirming a strong behavioural benefit of spatial‐temporal predictability, we show lateralisation of beta oscillations in anticipation of the response associated with the upcoming target location and show that this also aligns to the expected timing of these forthcoming events. This effect was found both when comparing between repeated (learned) and new (unlearned) sequences, as well as when comparing targets that were expected after short vs. long intervals within the repeated (learned) sequence. Our findings suggest that learning of spatial‐temporal structure leads to proactive and dynamic modulation of motor cortical excitability in anticipation of both the location and timing of events that are relevant to guide action.

## Introduction

Many actions in daily life consist of structured sequences. Examples can be found in speech, driving a car, performing sports or playing the piano. As sequences are repeated, performance improves. In the laboratory, sequence learning is typically studied using the serial reaction time (SRT) task (Nissen & Bullemer, 1987), in which the order of sequence elements associated with predefined actions is repeated. Typically, visual stimuli in particular spatial locations are associated with particular button presses.

The vast majority of SRT studies to date have focused on the learning about the spatial structure of events (see Abrahamse *et al*., 2010 and Schwarb & Schumacher, 2012 for reviews). They show that performance improves over the course of the experiment, while the precise sequence information (and the fact that elements are repeated) often remains implicit. Moreover, naturally occurring sequences often also contain temporal structure. Accordingly, a growing number of studies reveal strong benefits to performance when both the spatial and temporal structures of the sequence repeat (Shin & Ivry, 2002; Karabanov & Ullen, 2008; O'Reilly *et al*., 2008; Kornysheva *et al*., 2013; Kornysheva & Diedrichsen, 2014; Sanchez *et al*., 2015).

Studies to date largely overlook a fundamental complementary question about sequence learning: How are learned spatial and temporal sequences utilised by the brain to improve performance? Learned sequences afford spatial and temporal predictions about the locations and timings of upcoming elements, which in principle could be used to modulate preparatory neural activity to enhance performance.

Studies in which predictions regarding upcoming sensory input or required motor responses are explicitly cued typically show anticipatory power decreases in alpha and beta oscillations over relevant (contralateral) sensory and/or motor areas (e.g. Pfurtscheller & Lopes da Silva, 1999; Sauseng *et al*., 2005; Schoffelen, 2005; Alegre *et al*., 2006; Thut *et al*., 2006; Kelly *et al*., 2009; Rihs *et al*., 2009; Gould *et al*., 2011; Jenkinson & Brown, 2011; Van Ede *et al*., 2011). Anticipatory decreases over sensory areas are thought to reflect increased excitability states that benefit the processing of the anticipated stimuli, while power decreases over motor areas are thought to reflect enhanced motor readiness. Studies investigating the additional influence of temporal expectations further show that the time course of such power modulations in the alpha and beta frequency bands adapts to the timings used in the task, thereby ensuring optimal preparation for the moment that targets are expected (e.g. Schoffelen, 2005; Alegre *et al*., 2006; Rohenkohl & Nobre, 2011; Van Ede *et al*., 2011).

We hypothesised that the hidden spatial and temporal predictions within complex visual‐motor sequences in an SRT setting would support similar anticipatory changes in oscillatory power. In SRT settings that manipulate spatial and temporal sequences, there is evidence for a strong behavioural interaction between space and time for improving performance (O'Reilly *et al*., 2008; for further evidence for such spatial‐temporal ‘synergies’, see also Doherty *et al*., 2005; Rohenkohl *et al*., 2014). We thus expected that anticipatory changes in oscillatory power would adhere to both the learned spatial and temporal aspects of the sequence, that is would not only lateralise depending on the predicted target/response, but also adapt to its expected timing.

Here, we used magnetoencephalography (MEG) to investigate these neural dynamics within visually guided action sequences. Whole‐head MEG measurements enabled us to evaluate the respective contributions of anticipatory neural dynamics in sensory and motor cortices. This is of interest, as both perceptual preparation and motor preparation have been argued to contribute to performance improvements in SRT tasks (Howard *et al*., 1992; Willingham, 1999; Hoffmann *et al*., 2003; Clegg, 2005; Deroost & Soetens, 2006; Song *et al*., 2008; Schwarb & Schumacher, 2012).

## Methods

### Participants

The study was approved by the Central University Research Ethics Committee of the University of Oxford (MSD‐IDREC‐C2‐2014‐036), and the study was conducted in compliance with the Declaration of Helsinki. All participants gave written informed consent. Twenty‐one young, healthy adults (aged 24.7 ± 3.9 (SD), nine males) completed the study. All were right handed according to self‐report, and all had normal or corrected‐to‐normal vision. Participants were paid £10 per hour. Each participant completed three experimental sessions on separate days: a behavioural session, followed by a magnetoencephalography (MEG) session taking place 1 or 2 days later, and a magnetic resonance imaging (MRI) session within 2 weeks after the MEG session was completed. The behavioural results across all three sessions have previously been published elsewhere (see Heideman *et al*., 2017). The current manuscript will focus on the data acquired in the MEG session only.

Three participants were excluded from the MEG analysis. One participant was excluded because of extreme fatigue, causing a high number of mistakes (percentage correct was smaller than the mean minus three times the standard deviation across participants). Another participant was excluded because of a very slow mean reaction time (larger than the mean plus three times the standard deviation across participants). A third participant was excluded due to technical problems during part of the data collection, resulting in loss of trigger information. Results of eighteen participants (aged 24.7 ± 4.0 (SD), eight males) were therefore included in the final MEG data analysis, on which we report. Because the results from the latter participant could be included in our previous publication of the behavioural data alone (Heideman *et al*., 2017), we note that the behavioural results are numerically different, although the pattern of results is equivalent.

### MEG and visual stimulation set‐up

Whole‐head MEG recordings were acquired in a magnetically shielded room with normal illumination using a 306‐channel Elekta NeuroMag MEG System (Elekta, Stockholm, Sweden) at the Oxford Centre for Human Brain Activity. A magnetic Polhemus FastTrak 3D system (VT, USA) was used for head localisation. Relative positions of three anatomical landmarks (nasion, left and right auricular points) were measured in addition to relative positions of four head position indicator coils.

MEG data were collected in three separate recordings of 10–12 minutes each. During the short breaks in between, the data were saved while participants remained seated in the MEG chair. MEG data were sampled at 1000 Hz using a 0.03–300 Hz bandpass filter during digitisation of the signal. ECG and horizontal and vertical EOG were recorded. Eye movements were additionally recorded with a video‐based eye tracker at 1000 Hz (EyeLink 1000; SR Research, ON, Canada). A four‐button bimanual fibre‐optic response device was used to collect manual responses.

Stimuli were created with MATLAB (MathWorks, Natick, MA, USA) and presented using Psychtoolbox version 3.0 (Kleiner *et al*., 2007). Stimuli were back projected (Panasonic PT D7700E, Panasonic, Osaka, Japan) on a 43 × 54.5 cm translucent screen placed 120 cm in front of the participant, with a spatial resolution of 1280 × 1024 and a refresh rate of 60 Hz.

### Experimental procedure and stimuli

**Figure 1:**
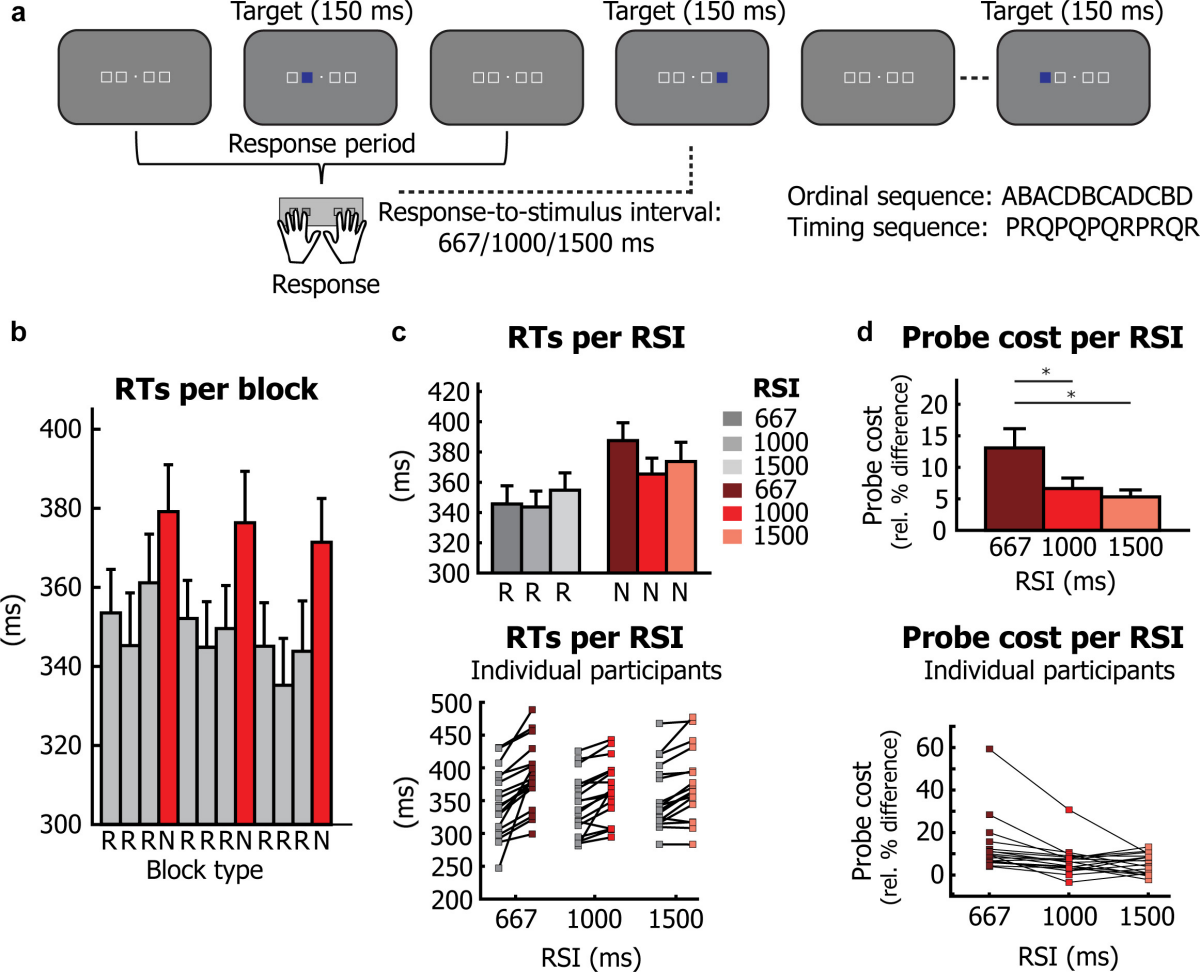
Task and behavioural results. (a) Four white squares displayed on a grey background outlined four possible target locations. Each target location corresponded to one of four buttons, to be pressed with the left and right middle and index fingers. Every time a blue target appeared in one of the four target locations, the corresponding button had to be pressed. Unknown to participants, both the order of targets and the order of response‐to‐stimulus intervals (RSIs) followed a repeating twelve‐element cycle. Three different RSI lengths were used: 667, 1000 and 1500 ms. Two different block types were presented: repeated sequence (R) blocks, containing eight repetitions of the twelve‐element cycle and new sequence (N) blocks in which a new spatial‐temporal sequence was presented. (b) Reaction times per block in the MEG session (prior to this session, incidental sequence learning had already been established in a behavioural session; see Heideman *et al*., 2017 for data showing the learning of the repeated sequence). Results for repeated sequence (R) blocks are shown in grey, while results for new sequence blocks (N) are shown in red. (c) Reaction times for each of the three RSI durations, separate for repeated sequence (R) and new sequence (N) blocks. The bottom plot reflects individual participant averages. (d) The probe cost was calculated as the relative difference between the average of all repeated sequence (R) and all new sequence (N) blocks, for each RSI length. The bottom plot shows results for each individual participant. Error bars present standard errors of the means (±1 SEM), calculated using the variance across participants. Asterisks indicate statistically significant effects.

The experimental task is shown in Fig. 1a. Participants performed a modified version of a serial reaction time (SRT) task in which they followed the order of targets presented on a screen by pressing the corresponding button on a button box, every time a target was presented. Four possible target locations were permanently indicated by white square outlines (2.12° × 2.12° of visual angle each; total width of all stimuli: 11.66° × 2.12° of visual angle). The squares were arranged horizontally, with two squares presented to the left and right of a small fixation square (0.17° × 0.17°of visual angle), against a grey background. Blue square targets were presented within these outlines.

Unbeknownst to participants, the positions of the targets (and therefore the corresponding responses) followed a twelve‐element cycle: ABACDBCADCBD, where A was the first position/left middle finger; B, the second position/left index finger; C, the third position/right index finger; and D, the fourth position/right middle finger. The next target was only displayed after a correct response was made; that is, participants had to correct themselves whenever they made a mistake. Three different response‐to‐stimulus intervals (RSIs) were used: 667, 1000 and 1500 ms. In addition to the repeated order of targets and responses, the order of RSIs also followed a repeating twelve‐element cycle: PRQPQPQRPRQR. The spatial and temporal sequences were therefore linked at the level of the twelve‐element cycle but otherwise unrelated.

After every three repeated sequence (R) blocks, a new, unlearned sequence was presented (N blocks). Twelve blocks (i.e. nine R blocks and three N blocks) were presented in total. To make sure sequence characteristics between R and N blocks were as similar as possible (see Reed & Johnson, 1994), new (unlearned) spatial‐temporal sequences were used instead of (more often used) pseudo‐random sequences. Each R block contained eight repetitions of the twelve‐element standard sequence. Within each N block, a new sequence was repeated eight times, but a different sequence was used for each of the three N blocks. The new spatial‐temporal sequence combinations used in N blocks were as follows: BDACBADCABCD/QPRPRPQRQPQR, CBDACDBADCAB/PRQRQRPQPRPQ, and DBCDCABDACBA/RQPQRPRPRQPQ. Each block contained 96 trials; therefore, 864 R trials and 288 N trials were presented in total. Eight‐second blinking breaks were inserted every 32 trials, with a longer break occurring after every four blocks. The first two trials after each break were excluded from the analysis (see Behavioural analysis). Each block therefore started at a different random point within the repeated sequence, to ensure approximately equal numbers of trial exclusions for each interval–position combination. The total session took approximately 45 minutes, including breaks. We note that despite our efforts to carefully match the new and repeated sequences, that because each sequence consists of combined spatial and temporal information, there might still be (small) structural or statistical differences between both conditions, based on interference from preceding sequence element–interval combinations. However, this is not a major concern, as our main contrast of interest is the short vs. long RSI *within* the repeated sequence, which concerns an (implicitly) learned spatial‐temporal association. Furthermore, we carefully constrained our analysis, to minimise the influence of the preceding trial (see Time‐frequency analysis).

### Behavioural analysis

Behavioural results for all three (behavioural, MEG and functional MRI) sessions have previously been described in Heideman *et al*. (2017). Our previous behavioural paper also included an analysis of assessment of awareness of order and temporal information, which was conducted after participants completed all three experimental sessions. These analyses indicated that participants were unaware of the temporal structure, but gained some intuition for the spatial pattern because they were able to reproduce parts of the sequence slightly above chance. In the current manuscript, we report the main RT results for the MEG session only. The behavioural data were analysed with MATLAB (MathWorks, Natick, MA, USA) and SPSS version 22 (IBM Corp. Armonk, NY, USA). As only combinations of three (triplets) or more stimuli were unique and allowed for preparation for the next, upcoming stimulus, the first two trials after each break were excluded from both the behavioural and the MEG analysis. We also excluded trials in which incorrect responses were given and trials with an RT shorter or larger than three times the mean plus or minus the SD of the participant's mean RT.

### MEG analysis

#### Pre‐processing and artefact rejection

MEG data analyses were performed using the in‐house OHBA Software Library (OSL) version 2.0, Fieldtrip (Oostenveld *et al*., 2011), and custom‐written MATLAB code. Using Maxfilter, MEG data from each participant were subjected to noise reduction using spatiotemporal signal space separation (TSSS). Neuromag MaxFilter software minimises extra‐cranial noise by separating signals arising from inside and outside the helmet (Taulu *et al*., 2004), with the temporal extension method additionally removing interference from nearby sources (Taulu & Simola, 2006). MaxFilter also compensates for the effect of head movement using continuous head position measurements. After using MaxFilter, the data were down‐sampled to 250 Hz. A 0.1‐Hz high‐pass filter was applied to the data to remove low‐frequency drift. Independent component analysis (ICA) was performed to reject artefacts associated with eye blinks, eye movements and heartbeat. We inspected all artifactual components visually before removing them from the data. After epoching the data, OSL's variance‐based automated artefact detection was applied. Trials excluded during the behavioural analysis stage, including the first two trials after each break (see Behavioural analysis), were excluded from the MEG data as well. On average 19.6 ± 4.6% of trials were excluded.

#### Region‐of‐interest selection based on event‐related fields

Bilateral motor and visual regions of interest (ROIs) were selected based on event‐related fields (ERFs) evoked by motor response and targets, thereby ensuring that ROI selection was independent of the main analysis period and signal features of interest (anticipatory time‐frequency data). Moreover, channel selection was performed independently of condition.

**Figure 2:**
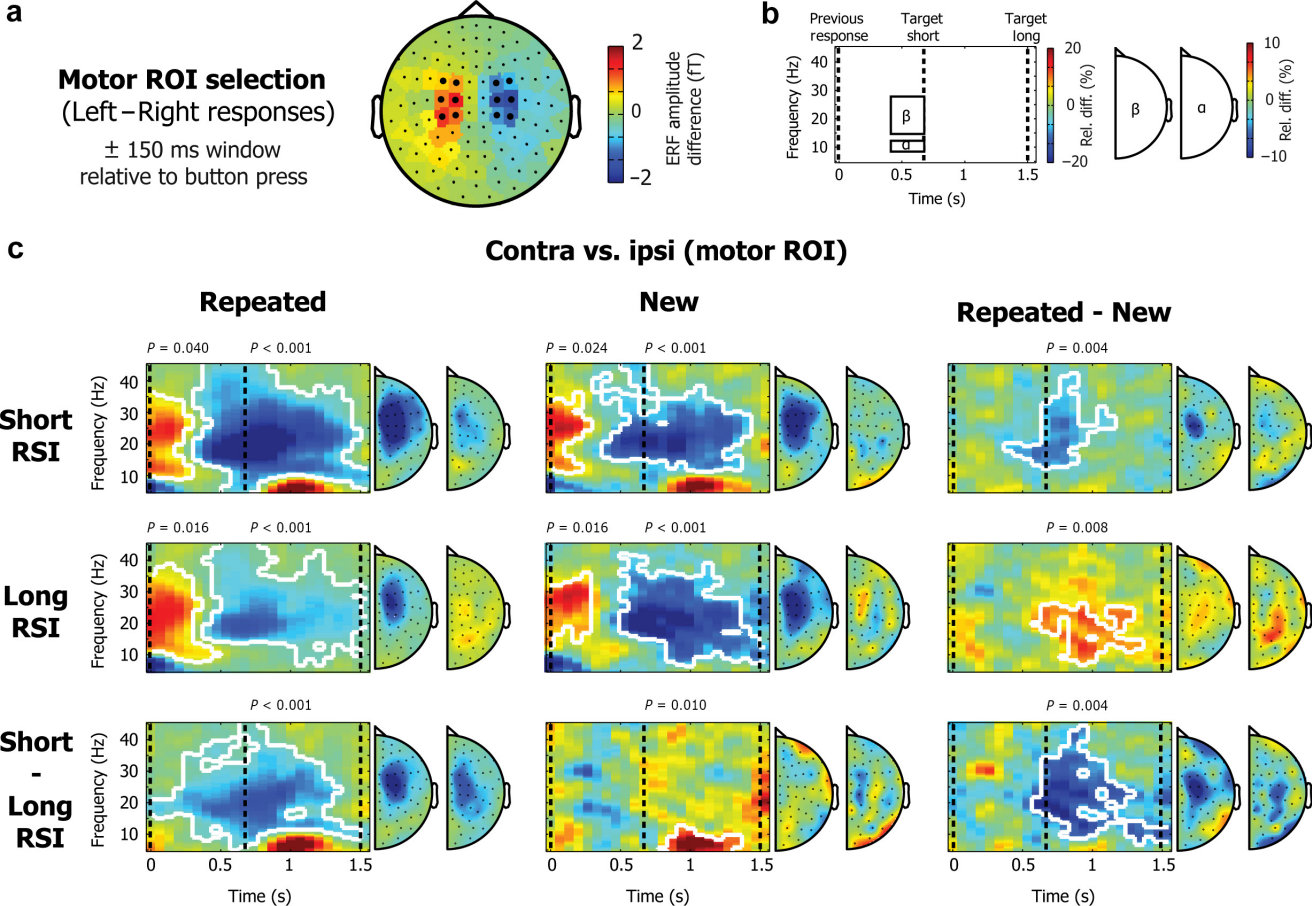
MEG results for the power difference between contralateral and ipsilateral motor channels. (a) Topography for the ERF difference between left vs. right motor responses in the ERF averaged for a 300‐ms window centred on the button press (collapsed across RSIs). The symmetrical left and right motor channels that were selected as the motor region‐of‐interest (ROI) in the MEG analysis are marked in black. (b) Schematic of the data depicted in c. TFR plots reflect frequencies between 5 and 45 Hz, locked to the preceding button press, which always occurred with the opposite hand, compared to the (anticipated) current target. Results are shown for targets that occurred either after a short RSI (667 ms) or after a long RSI (1500 ms), and were always preceded by a medium RSI. Topographies show averages for the beta (15 ‐ 28 Hz, shown on the left) and alpha (8–12 Hz, shown on the right) frequency bands, for a window between 400 and 667 ms after the button press (i.e. just before a ‘short target’ would be presented). (c) Time–frequency representation (TFR) plots for contra vs. ipsi ROI channels (relative to the target location and therefore response side), shown separately for the repeated sequence condition (first column), the new sequence condition (second column) and for the repeated vs. new contrast (third column). Rows depict results for the short RSI (top row), the long RSI (second row), and the difference between the short and the long RSI (bottom row). Significant clusters are outlined in white. Topographies similarly reflect results for contralateral vs. ipsilateral activity in symmetrical channel pairs (for convenience, plotted in the right channel element of each channel pair).

**Figure 3:**
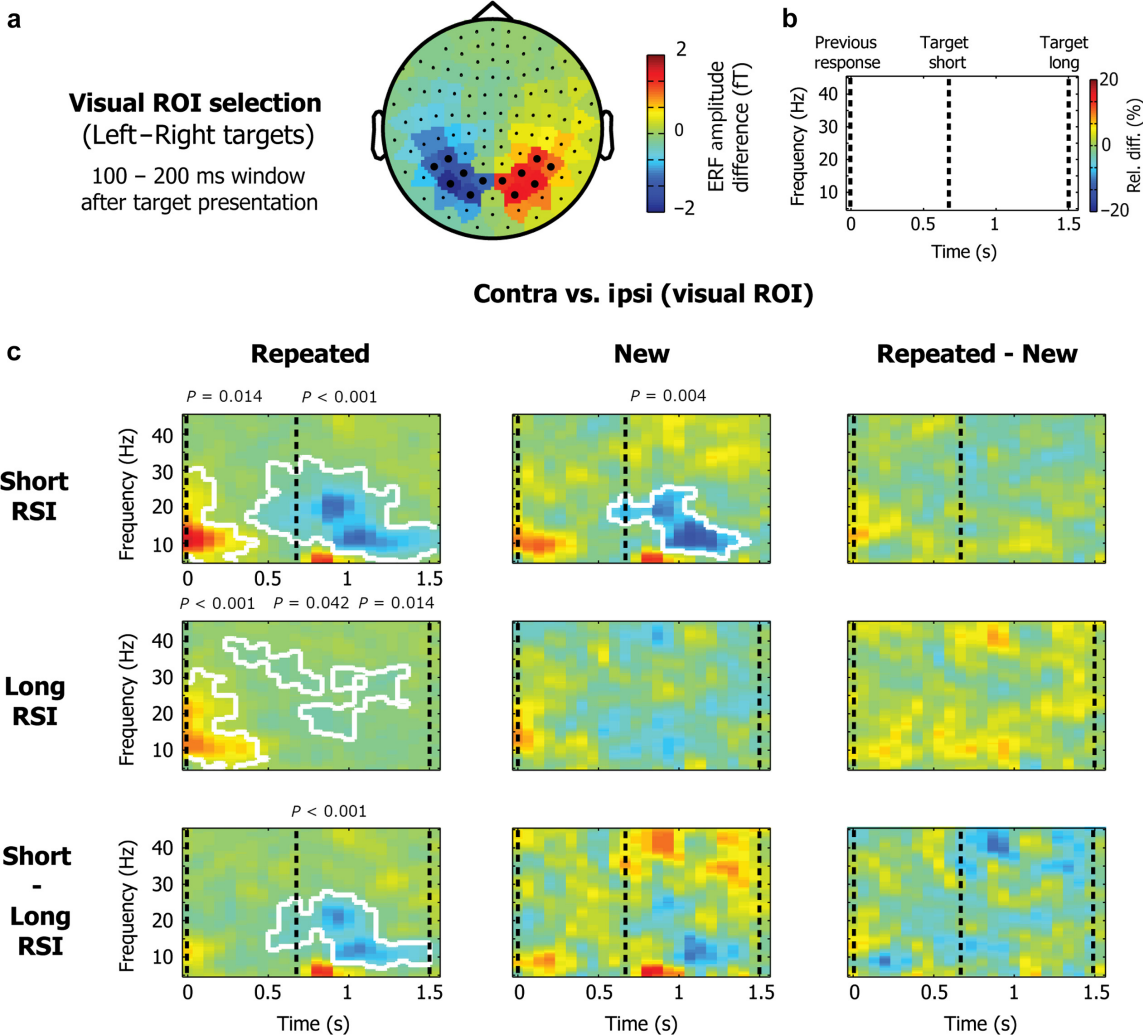
MEG results for the power difference between contralateral and ipsilateral visual channels. Same conventions as in Fig. 2, except all contrasts involve the difference between contra and ipsilateral visual ROIs (as opposed to motor ROIs). Visual ROIs were based on the ERF difference between left vs. right targets in the 100–200 ms window after target presentation, as depicted in panel a. Corresponding topographies for alpha and beta are shown in Fig. 2.

ERFs were calculated separately for left (positions 1 and 2) and right (positions 3 and 4) targets. Data for the planar gradiometer pairs were combined, resulting in a 102‐channel combined planar gradiometer map in sensor space. A left vs. right difference ERF was calculated for each participant separately and subsequently averaged across participants. Motor ROIs were selected based on the average left vs. right difference ERF topography in a window of 300 ms centred on the button press (see Fig. 2a). Based on the topography, six (symmetrical) channel pairs were selected on the left (MEG0412 + 0413, MEG0422 + 0423, MEG0432 + 0433, MEG0442 + 0443, MEG1812 + 1813, MEG1822 + 1823) and on the right (MEG1112 + 1113, MEG1122 + 1123, MEG1132 + 1133, MEG1142 + 1143, MEG2212 + 2213, MEG2222 + 2223). Visual ROIs were similarly selected based on the average left–right difference ERF topography in a window of 100–200 ms after target presentation (see Fig. 3a). We again selected six (symmetrical) channel pairs on the left (MEG1632 + 1633, MEG1642 + 1643, MEG1912 + 1913, MEG1922 + 1923, MEG1942 + 1943, MEG2042 + 2043) and on the right (MEG2032 + 2033, MEG2312 + 2313, MEG2322 + 2323, MEG2342 + 2343, MEG2432 + 2433, MEG2442 + 2443).

#### Time‐frequency analysis

Time‐frequency analysis was performed on Hanning‐tapered data using a short‐time Fourier transform for frequencies between 4 and 45 Hz (in 0.5 Hz steps). A fixed sliding time window of 300 ms was advanced over the data in steps of 67 ms. Power time series in planar gradiometer pairs were combined (Cartesian sum), resulting in a 102‐channel combined planer gradiometer map in sensor space.

Because each button press was immediately followed by the start of the following trial, no clean baseline period was present in the data. All data were therefore explored by looking at power differences between contralateral and ipsilateral ROIs only. For each ROI, we computed relative contrasts between contralateral and ipsilateral expected visual targets ([[contra ‐ ipsi]/[contra + ipsi]]*100), and subsequently averaged across both ROIs. We also calculated this contra vs. ipsi contrast for each symmetrical channel pair (symmetrical along the midline) to obtain the corresponding contra vs. ipsi topography plots.

We took particular care to equate the influence of previous button presses on the current trial between conditions. Because of the nature of spatial‐temporal sequence learning, conditions differed with regard to the proportion of trials in which the previous response was made with the same or the opposite hand and differed with regard to the RSI on the previous trial. Effects relating to previous trials can influence both behaviour and brain activity (see, e.g. Van der Lubbe *et al*., 2004; Los, 2010), and can in principle introduce spurious findings. In order to avoid any effects related to different proportions of preceding responses or RSI, we limited our initial analysis to a subset of trials in which all relevant parameters were equated. We only analysed trials in which the previous response (depicted at time point 0 in Fig. 2 and Fig. 3) was on the opposite hand. In addition, we only analysed short and long RSI trials (667 and 1500 ms), where the RSI on the previous trial was of intermediate length (1000 ms). For R blocks, on average 118 ± 2 (SE) trials per RSI per participant were included, and for N blocks, this was 20 ± 0.4 trials per RSI per participant.

The main period of interest was the first 667 ms after the button press to the previous target (i.e. the time period corresponding to the short RSI). This is the time period during which spatial‐temporal expectations (induced by the sequence structure) differ between repeated and new sequences, and between short and long RSIs in repeated blocks. Differences between conditions were evaluated using Fieldtrip's nonparametric cluster‐based permutation tests, which circumvent the multiple comparisons problem by testing the full time‐frequency space under a single permutation distribution of the largest cluster (Maris & Oostenveld, 2007). We used 1000 permutations with a two‐tailed alpha level of 0.05.

#### Post hoc analyses

In addition to the main analysis described above, we performed two post hoc analyses on our data. First, we evaluated the relation between contra vs. ipsi beta power differences and our behavioural results (RTs) by investigating trial‐by‐trial Pearson's correlations for the short interval in the repeated condition for each time‐frequency point. We limited our analysis to the same subset of trials as described above. These results were evaluated using Fieldtrip's nonparametric cluster‐based permutation tests on the correlation values.

Secondly, to evaluate whether the beta suppression in our experiment might show a parametric effect, we conducted an additional analysis for the repeated sequence condition using all thee RSI lengths. We again only included trials where the previous response was on the opposite hand. For this analysis, it was necessary to relax the criterion that the previous RSI had to be of intermediate length. Contrasts were again calculated as relative contrasts (see above) and evaluated using Fieldtrip's nonparametric cluster‐based permutation tests. To quantify the influence of temporal expectations, we investigated magnitude (power) differences in the early interval after the previous sequence element, with the rationale that when targets are expected early (as compared to later), power modulations should be more pronounced in the early interval. Therefore, in addition, we extracted the data of interest in an *a priori* defined pre‐target window (i.e. beta band activity [15–28 Hz] in the window immediately preceding the first possible target [400–667 ms after the previous response]) to directly compare contra vs. ipsi differences between conditions in a repeated‐measures ANOVA.

## Results

Participants took part in a multisession experiment involving a spatial‐temporal SRT task. A previous behavioural publication (Heideman *et al*., 2017) describes the behavioural results for all three (behavioural, MEG and fMRI) sessions. In the current manuscript, we report results for the MEG session only and focus on anticipatory neural dynamics related to the utilisation of incidentally acquired combined spatial and temporal sequences.

### Behavioural results

RTs as a function of block type and RSI are shown in Fig. 1b. Learning in SRT tasks can be expressed in two ways. First, learning is often shown by a decrease in RTs over the course of the first block(s) of the experiment (see Heideman *et al*., 2017; for the learning results for the behavioural session preceding the MEG session). In addition, learning in SRT tasks can be shown by introducing a new or random sequence instead of the usual, repeated sequence. In this study, we did this in three blocks (red bars, Fig. 1b). Paired‐samples *t*‐test confirmed that RTs in blocks with new sequences (new blocks; N) were larger than RTs in blocks with the standard, repeated sequence (repeated blocks; *R*;* t*(17) = 5.35, *P* < 0.0001), indicating that participants utilised the learned spatial‐temporal sequence structure in the R blocks.

To establish the specific utilisation of the temporal aspects of the learned sequence structure, we investigated differences in ‘probe costs’ (quantified as the difference between N vs. R blocks) for the different RSIs that were used in the task (667, 1000 and 1500 ms). Figure 1c and d shows the RTs and probe costs as a function of RSI, with group averages shown at the top, and results for individual participants at the bottom. A repeated‐measures ANOVA with the factors RSI (667, 1000 or 1500 ms) and block type (R or N) revealed main effects of both RSI (*F*_2,16_ = 10.72, *P* = 0.001) and block type (*F*_1,17_ = 34.25, *P* < 0.0001), and, importantly, an interaction between RSI and block type (*F*_2,16_ = 10.92, *P* = 0.001). This interaction between RSI and block type is best appreciated by looking at the probe cost as a function of the different RSIs. Probe costs differed significantly between RSIs (*F*_2,16_ = 8.02, *P* = 0.004), with follow‐up *t*‐tests showing that probe costs for the short RSI were larger than probe costs for the medium RSI (M ± SE = 43 ± 8 ms vs. 23 ± 6 ms; *t*(17) = 4.95, *P* = 0.0001) and the long RSI (M ± SE = 43 ± 8 ms vs. 19 ± 4 ms; *t*(17) = 3.04, *P* = 0.007. Benefits in response times for the medium and long RSI conditions did not differ (M ± SE = 23 ± 6 ms vs. 19 ± 4 ms; *t*(17) = 0.71, *P* = 0.490).

These data suggest that the largest behavioural benefit from the learned spatial‐temporal sequence is obtained for targets occurring after a short RSI. This is in line with the typical observation in cued temporal orienting tasks (Coull & Nobre, 1998; Miniussi *et al*., 1999; Correa *et al*., 2006; Nobre, 2010; Rohenkohl *et al*., 2014) in which the beneficial effect of valid temporal foreknowledge is most pronounced at the short interval, but is much weaker at the later interval as a result of concurrent hazard rate‐based expectations (see Heideman *et al*., 2017 for further discussion). Based on these data, we looked for anticipatory neural dynamics that would adhere to a similar pattern, with the strongest anticipatory modulations occurring early after each response when the next target is also expected early (i.e. at the short RSI).

After the final experimental session, we assessed to what degree the spatial and temporal sequence information remained implicit to participants (see Heideman *et al*., 2017 for a full description of the assessment and results). Our assessment of awareness of spatial (spatial) sequence revealed that, although participants did not generally believe they gained explicit knowledge about the repeated order (confidence ratings were low), they were able to perform a sequence generation and prediction of next target task slightly above chance. This was, however, not the case for the temporal structure. In addition to very low confidence ratings, participants performed at chance level when asked to reproduce the temporal structure of the task. All temporally specific modulations (including interactions between incidentally acquired temporal and spatial structure) are therefore considered to reflect implicit learning in this study.

### MEG results

#### Motor preparation

Left and right motor ROIs (Fig. 2a) were selected based on the topography of ERFs evoked by left vs. right responses in a 300‐ms window centred on the button press. We used these ROIs to extract anticipatory contra‐ and ipsilateral motor activity.

Figure 2c shows the time‐ and frequency‐resolved normalised power differences between contralateral and ipsilateral ROI channels (contra vs. ipsi), as a function of block type (columns) and RSI (rows). Data are locked to the previous button press, such that the expected target and motor response (in repeated blocks) occur either early or late after this event. Contra‐ and ipsilateral ROIs are defined relative to the upcoming response (which was always opposite to the previous response; see Time‐frequency analysis). Topographies mark the same contra vs. ipsi difference (evaluated for each symmetrical channel pair), averaged over the indicated time‐frequency windows depicted in Fig. 2b. Statistical testing was performed on the time‐frequency matrices using cluster‐based permutation statistics (Maris & Oostenveld, 2007). Significant clusters are outlined in white.

In both the repeated and the new blocks, for both the short and the long RSI, we observed a clear modulation of power that was largely restricted to the beta band. Beta power in the first 200 ms after the response (time point 0 in Fig. 2c) was larger contralateral than ipsilateral (repeated short: two‐sided cluster *P* = 0.044; repeated long: two‐sided cluster *P* = 0.014; new short: two‐sided cluster *P* = 0.036; new long: two‐sided cluster *P* = 0.014), followed by a prolonged period with less power contralateral than ipsilateral (two‐sided cluster *P* < 0.001 for all four conditions). We must immediately point out that this is most likely a consequence of our analysis pipeline, in which we only included trials in which the previous response was made with the opposite hand compared to the upcoming response. Instead, we were particularly interested in the differences in this modulation when the expected RSI was short or long (in repeated blocks) and between repeated and new trials (particularly for the short RSI, given our behavioural results). Critically, these contrasts of interest are not confounded by the previous response, because both the previous response side (always the opposite hand) and the previous RSI (always the intermediate RSI) were carefully equated in these four conditions.

We first considered the difference between short and long RSI trials within repeated sequences (Fig. 2c, left column, bottom panel). Based on our behavioural results, we hypothesised that participants would show a stronger engagement of their motor system (as reflected in lower contralateral, relative to ipsilateral, alpha and beta power) early after their response, when the next target/response was expected to follow after a short, compared to a long RSI. In accordance with this, we observed a significant cluster (two‐sided cluster *P* < 0.001), revealing a stronger lateralisation (less power contralateral than ipsilateral) in short compared to long RSI trials. This effect was most prominent in the beta band and started well ahead of the anticipated target/response. The topography of this modulation was centred on the selected motor channels (indicating an origin in motor or pre‐motor cortices), and this was the case for modulations in both the beta and the alpha band.

In contrast to these results, and according to our predictions, no significant short vs. long RSI difference was observed for the new blocks (Fig. 2c, middle column, bottom panel). In new blocks, participants could not predict the upcoming RSI. Moreover, the contrast of the RSI effects between repeated and new blocks (third column, bottom panel) showed that the interaction between RSI and block type also survived statistical testing (two‐sided cluster *P* = 0.006).

This pattern of results was replicated in the complementary contrast between repeated and new blocks, when considering the short RSI (Fig. 2c, right column, upper panel; two‐sided cluster *P* < 0.001). The topography of this contrast was again centred on the selected motor channels and seemed particularly pronounced in the beta band.

#### Visual anticipation

Because the spatial aspects of the task involved both a lateralised visual stimulus and a related lateralised motor response, one might expect that the utilisation of the learned spatial‐temporal sequence structural might additionally modulate anticipatory perceptual dynamics in visual sites. Putative concurrent neural dynamics of visual anticipation would be expected to be most pronounced in the alpha band (Worden *et al*., 2000; Sauseng *et al*., 2005; Thut *et al*., 2006; Wyart & Tallon‐Baudry, 2008; Kelly *et al*., 2009; Jensen & Mazaheri, 2010; Gould *et al*., 2011). Yet, the alpha‐based topographies in Fig. 2c revealed a largely motor origin of the anticipatory dynamics. To assess further the potential dynamics associated with visual anticipation, we additionally repeated the analysis performed over contralateral and ipsilateral motor channels with contralateral and ipsilateral visual ROIs.

Left and right visual ROIs (Fig. 3a) were selected from the left vs. right target topography in a window 100 to 200 ms after target presentation and were used to extract anticipatory contra‐ and ipsilateral visual activity. Time‐frequency plots of the normalised power differences between contra and ipsi ROI channels (Fig. 3c) are again shown as a function of block type (rows) and RSI (columns). Data are similarly locked to the previous button press, with the upcoming target/response occurring on the opposite side. Upcoming targets were again presented either after a short (667 ms) or long (1500 ms) RSI.

In contrast to the motor ROI results, in the visual ROIs differences in anticipatory activity between conditions were less convincing. Only the difference between the short and long RSI for repeated trials survived statistical comparison (two‐sided cluster *P* < 0.002), but this difference could not convincingly be attributed to an anticipatory effect. Indeed, when inspecting the alpha topographies in Fig. 2c it is clear that (at least) the pre‐target activity in this cluster may include a bleed through from more central (pre‐) motor areas, where this anticipatory modulation by expected target/motor timing is most pronounced.

#### Trialwise correlations with reaction time

**Figure 4:**
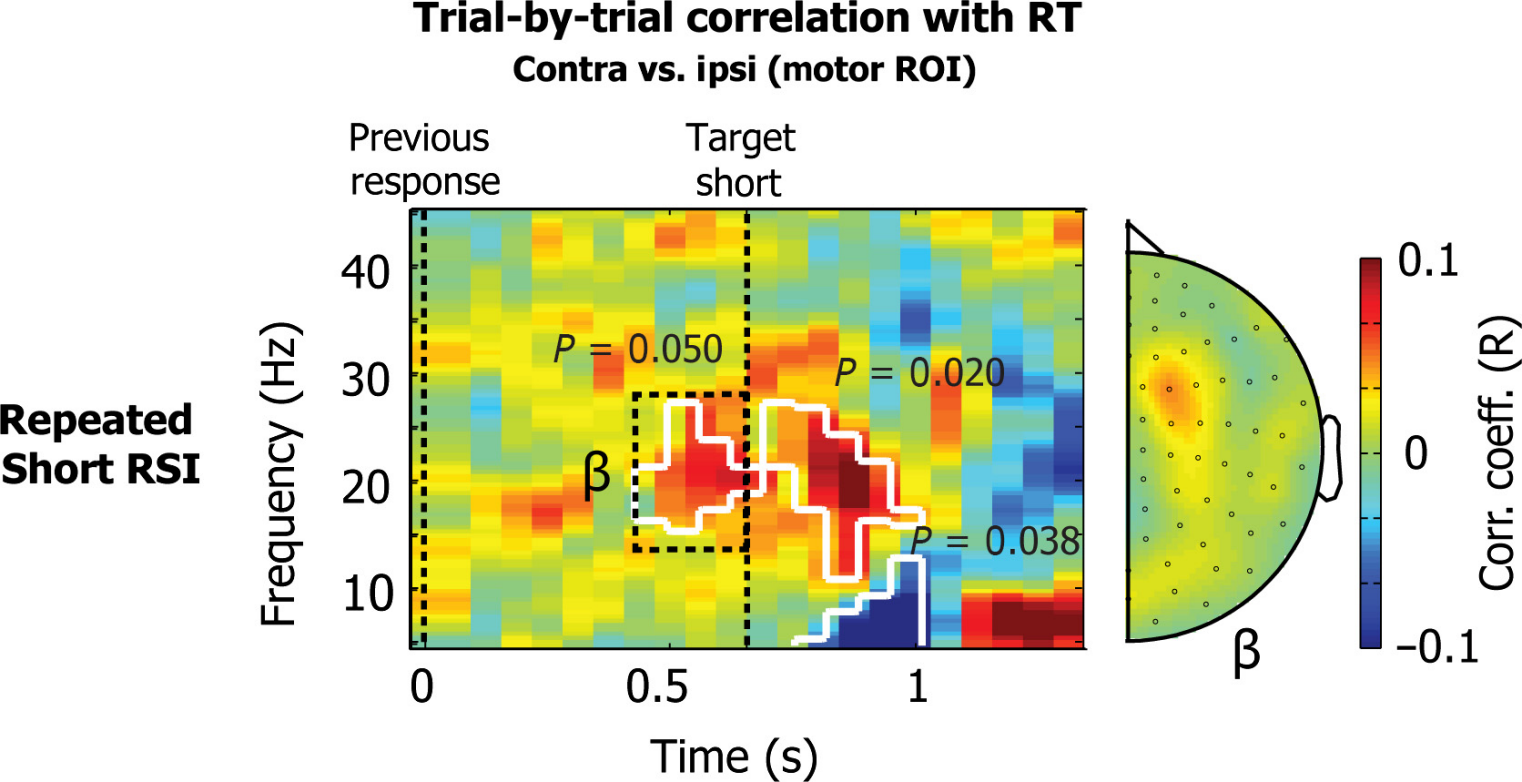
Trial‐by‐trial correlations between power and RT for the short RSI (667 ms) in the repeated condition. The data show the Pearson correlation coefficient for each time‐frequency point between 5 and 45 Hz, for the contra vs. ipsi contrast, locked to the previous button press. Data are plotted for the motor ROI channels shown in Fig. 2a. The topography shows the average for the beta (15–28 Hz) frequency band, for a window between 400 and 667 ms after the button press (i.e. just before the presentation of the short target). Significant clusters are outlined in white. The topography reflects results for contralateral vs. ipsilateral activity in symmetrical channel pairs.

To investigate whether the contra vs. ipsi beta power difference was indeed associated with improved performance, we performed a post hoc analysis of the correlation between power and RTs, focusing on short RSI trials in repeated blocks. If it is such that lower contra vs. ipsi beta power (putatively reflecting stronger expectation) confers an RT benefit (i.e. lower RT), then this should show as a positive correlation. For this purpose, we calculated the trial‐by‐trial Pearson correlation for each time‐frequency point. Results are shown in Fig. 4, with significant clusters outlined in white. These results clearly show a significant correlation both pre‐ and post‐target (two‐sided cluster *P* = 0.050 pre‐target and two‐sided cluster *P* = 0.020 post‐target), indicating that participants responded more quickly when there was a larger contra vs. ipsi difference in beta power. In addition, RTs correlated negatively with post‐target low‐frequency power (putatively, the ERF; *P* = 0.038). The topography of this pre‐target effect (the effect in which we were particularly interested) again confirmed a localisation to the (pre‐)motor areas.

#### Beta modulations in the medium interval

**Figure 5:**
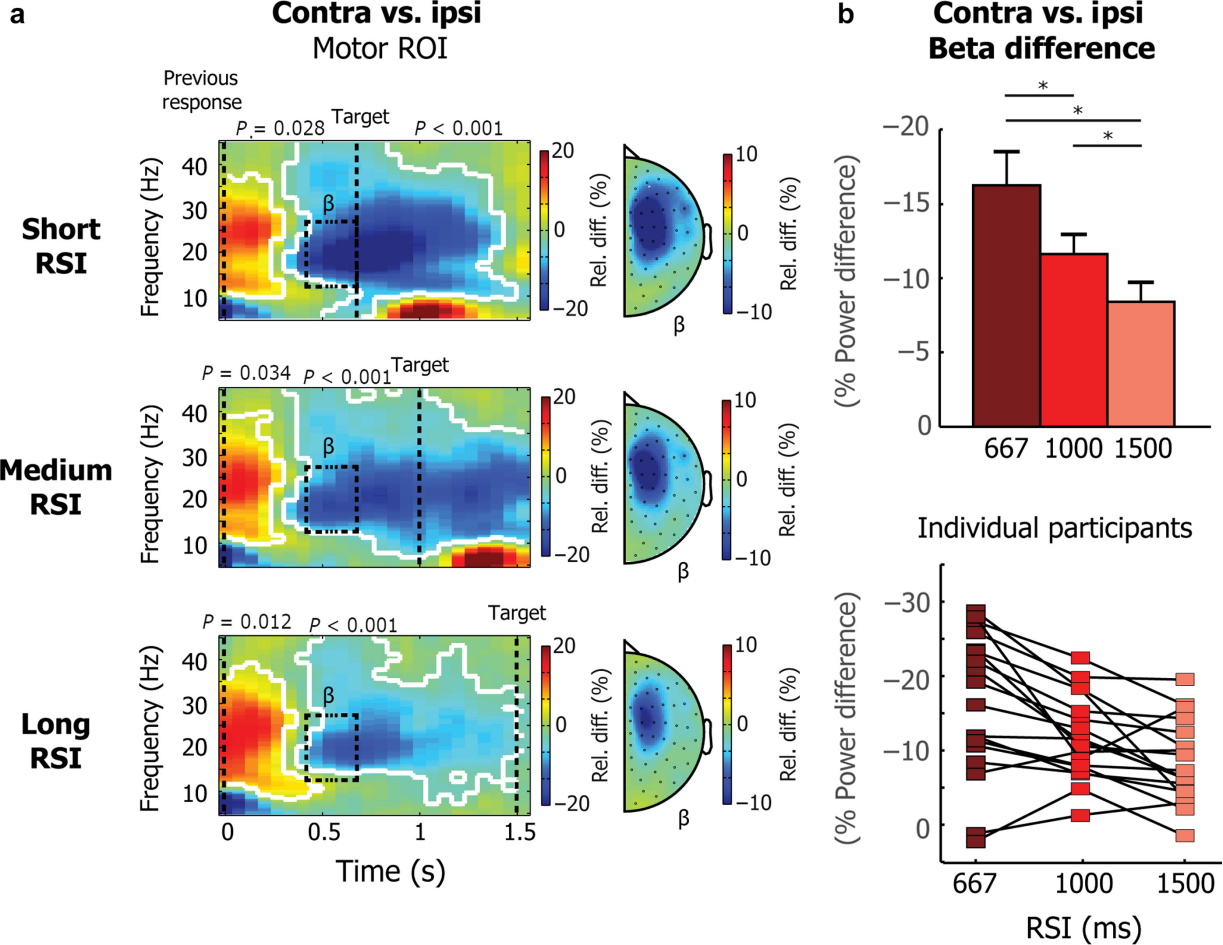
Beta power differences between contralateral and ipsilateral motor channels for all three RSIs. (a) TFR plots reflect frequencies between 5 and 45 Hz, locked to the preceding button press, which always occurred with the opposite hand, compared to the (anticipated) current target. Data are shown for the motor ROI channel selection shown in Fig. 2a. Results are shown for targets that occurred either after a short RSI (667 ms; top plot), medium RSI (1000 ms; middle plot) or a long RSI (1500 ms; bottom plot). Significant clusters are outlined in white. Topographies show averages for the beta (15–28 Hz) band, for a window between 400 and 667 ms after the button press. (b) Beta power difference between contralateral and ipsilateral motor channels for the same time and frequency selection as used for the topographies in a. The bottom plot shows individual participant data. Asterisks indicate statistically significant effects.

To evaluate whether beta modulations in our experiment varied parametrically according to the learned RSI, we reran our analysis for the repeated condition using all three RSI lengths. Data are shown in Fig. 5, for the same motor ROIs that were selected in Fig. 2. Figure 5a follows the same convention as Fig. 2, except that it also includes data from the medium RSI condition (note that for this analysis, we had to relax the constrain that the previous RSI should be the medium RSI).

Figure 5b (top) shows the group average of the contra vs. ipsi beta power difference for the same time‐frequency window as used for the topographies in Fig. 5a, as well as Fig. 2. Results for individual participants are shown at the bottom. A repeated‐measures ANOVA showed a significant effect of RSI (*F*_2,16_ = 12.08, *P* < 0.0001). Follow‐up *t*‐tests showed that the contra vs. ipsi beta power difference (here plotted as a positive effect) was significantly larger for the short, compared to the medium (*t*(17) = 3.24, *P* = 0.005) and long RSI (*t*(17) = 5.02, *P* = 0.0001) and also differed significantly between the medium and long RSI (*t*(17) = 2.94, *P* = 0.009). These results show that the strength of beta modulation differed depending on temporal expectation in a parametric fashion: the earlier a target is expected, the stronger the beta modulation early after the preceding sequence element.

## Discussion

We set out to investigate anticipatory oscillatory dynamics related to the use of incidentally acquired, hidden, spatial‐temporal sequences. Behavioural data suggest that predictions in learned spatial‐temporal sequences are used proactively and dynamically to enhance performance. Similar to the effects in spatial‐temporal orienting studies using explicit cues, performance benefits in our SRT task were strongest for short (vs. medium and long) intervals (see Heideman *et al*., 2017 for elaboration). In line with these results, our MEG data revealed anticipatory power modulations in the beta band (lower contra‐ vs. ipsilateral power) that adapted in a parametric fashion to the expected location and timing of elements within the visual‐motor sequence. This difference was observed when comparing the short RSI for learned (repeated) vs. unlearned (new) sequences (i.e. ‘early’ vs. ‘no’ expectation), as well as when comparing short and long RSIs for the repeated sequence (i.e. ‘early’ vs. ‘late’ expectation). We further show that responses to elements that were expected after a short preceding interval were faster in trials in which beta was more suppressed in contralateral (relative to ipsilateral) motor cortical sites – suggesting a role for these anticipatory beta modulations in mediating the observed performance benefits.

These results advance the literature in two key ways. First, they complement the motor sequence‐learning literature, by focusing on neural dynamics of sequence *utilisation* as opposed to sequence learning. Second, they reveal that typical behavioural and EEG/MEG patterns observed with explicit spatial and temporal cueing generalise to settings involving complex, incidentally acquired sequences. By doing so, they reveal that canonical neural dynamics of motor preparation flexibly adapt to learned spatial and temporal sequence structure, even when this structure remains largely implicit and even when interelement intervals are variable (i.e. non‐rhythmic). We elaborate below.

### Neural dynamics of the utilisation of predictive spatial‐temporal sequence structure

Most prior neuroimaging work investigating the neural basis of behavioural performance benefits associated with incidentally acquired sequence structure (typically in SRT tasks) focused on the learning of such sequence information. Moreover, most of these studies only investigated this for spatial structure. Complementing this work, we were particularly interested how, once such sequence structure has been acquired, this learned information (in our case both spatial‐ and temporal structure) is subsequently used to optimise behaviour.

Hemodynamic imaging techniques like fMRI have been useful for revealing areas and networks involved in learning in SRT tasks (e.g. Schendan *et al*., 2003; Gheysen *et al*., 2010; Hardwick *et al*., 2013) and for studying how spatial and temporal sequence features are represented (Kornysheva & Diedrichsen, 2014; Bednark *et al*., 2015). MRI is a useful tool for looking at brain areas involved in learning, which generally happens on a slow timescale, over the course of several blocks. However, it is not well suited for the investigation of fast temporal dynamics, which is essential when studying how these representations are used to guide behaviour, while sequences are unfolding. By turning to high‐temporal resolution MEG measurements, we were able to study precisely this.

Although electrophysiological methods have been used to study learning in SRT tasks, these too have focussed primarily on the acquisition of spatial structure. An EEG study looking at lateralised readiness potentials within learned visual‐motor sequences also found that participants showed initial activation of incorrect (but expected) responses when they were presented with a deviant item (Eimer *et al*., 1996). Pollok *et al*. (2014) observed a stepwise decline of alpha desynchronisation associated with motor learning. Furthermore, in their study the improvement of reaction times varied linearly with the amount of beta desynchronisation, particularly during consolidation. Another recent study reported a reduction in alpha and theta power over posterior areas with learning for sequenced blocks (compared to random blocks), together with a reduction in alpha‐to‐low‐gamma phase–amplitude coupling over right parietal and bilateral frontal areas (Tzvi *et al*., 2016). The authors suggest that the reduced phase–amplitude coupling reflects a shift away from visually guided motor selection, towards implementation of the learned motor sequence. This study, however, did not look at beta activity.

The present study complements these studies in three key ways. First, it looks at power modulations reflecting the utilisation of the incidentally learned structure, rather than at the learning per se. Second, it looks at dynamic changes in neural activity at a much finer timescale, associated with spatial and temporal contingencies between successive sequences elements rather than changes in the state of brain activity over extended blocks of learning. Third, it considers both the temporal and spatial structure of sequences, showing that preparation is specific to precise target–interval combinations.

### Anticipatory spatial‐temporal beta modulations in hidden spatial‐temporal sequences

The main finding in the present study is that anticipatory power modulations in the beta band adapt to predictive sequential structure, with both spatial and temporal specificity. The effects point to proactive utilisation of spatial and temporal predictions embedded in sequences, in a way that resembles the use of explicit cues about the location and timing of upcoming targets or motor responses. It is well known that motor (and somatosensory) preparation is associated with attenuated alpha and beta power over corresponding motor and sensory regions, both when preparing voluntary movements and in anticipation of task‐relevant targets (Jasper & Penfield, 1949; Pfurtscheller & Lopes da Silva, 1999; Jones *et al*., 2010; Van Ede *et al*., 2010, 2011; Jenkinson & Brown, 2011; Kilavik *et al*., 2013). Moreover, an increasing number of studies have shown that these modulations are not only sensitive to spatial expectations (showing larger attenuation in contralateral vs. ipsilateral sites; e.g. Nagamine *et al*., 1996; Taniguchi *et al*., 2000; Doyle *et al*., 2005; Alegre *et al*., 2006; Jurkiewicz *et al*., 2006; Zhang *et al*., 2008; Van Ede *et al*., 2011, 2010), but also to temporal expectations (e.g. Schoffelen, 2005; Alegre *et al*., 2006; Van Ede *et al*., 2011). If you know that a target requiring a response will appear shortly, beta power decreases earlier than when this target is expected only later, similar to the difference between short, medium and long RSIs for the repeated sequence in the current study. Interestingly, interval timing in perceptually guided motor tasks has been reported to be encoded by neurons in medial pre‐motor cortex (e.g. Merchant *et al*., 2013) which may provide the possible source for instantiating the temporal alignment of the anticipatory beta modulation reported here.

Previous studies using regular, isochronous rhythmic sequence have also shown modulation of oscillatory power (e.g. Praamstra *et al*., 2006; Heideman *et al*., 2015). These studies also show that temporal regularities can modulate neural excitability in the absence of explicit temporal cues, showing that that beta power starts to decrease earlier in a faster, compared to a slower rhythmic stream. However, in these studies the spatial (motor) element was random, and therefore, motor preparation was spatially (effector) unspecific.

Our demonstration of spatially and temporally specific modulation of motor preparation in complex spatial‐temporal sequences complements findings using rhythmic sequences in two important ways. Our results show that proactive and flexible temporal modulation of brain activity by an implicit temporal structure does not require a simple, constant rhythm. Isochronous rhythms have been proposed to be able to align to endogenous, naturally occurring brain rhythms to regulate neural excitability in a rhythmic fashion (Schroeder & Lakatos, 2009; Schroeder *et al*., 2010). Although this may indeed be a potent mechanism for modulating excitability to enhance performance to naturally occurring rhythmic stimulation (e.g. Lakatos *et al*., 2008, 2009; Stefanics *et al*., 2010; Besle *et al*., 2011; Cravo *et al*., 2013), this would not be sufficient to support the effects observed in the context of our more complex spatial‐temporal sequence. Our temporal sequence only repeated every 12 elements, and the specific intervals were not in a harmonic relation to one another.

The second intriguing and important aspect of our results is that temporal modulations following the hidden sequential structure of the task were spatially specific. This occurred despite the fact that the spatiotemporal contingencies were complex, with temporal‐motor associations being specific to the different positions across the twelve items of orthogonal spatial and temporal sequences. Complementary studies looking specifically at interactions between spatial and temporal information have shown that such combined effects are especially powerful (i.e. ‘super‐additive’; Doherty *et al*., 2005; O'Reilly *et al*., 2008; Rohenkohl *et al*., 2014), in comparison with either type of information in isolation. Following the strong synergy observed between spatial and temporal sequences in behavioural effects (O'Reilly *et al*., 2008), we hypothesise that the temporal predictions in our task strongly potentiate the modulation of anticipatory motor preparation. It will be interesting to verify this in future studies by comparing markers of motor preparation in sequences with both spatial and temporal structure to sequences with either feature in isolation. Note that the current task does not allow us to make such a distinction, because both spatial and temporal sequences were always present, that is, we can only establish an effect of spatial‐temporal expectations. Another limitation of the current task is that due to our trial selection procedure, only a small number of trials (i.e. 20 per RSI) could be included for the new sequence conditions. At the same time, we note that we find an effect despite this limited number and, moreover, that our main contrast of interest (repeated short vs. long) included 118 trials per RSI.

### The nature of proactive anticipation in SRT tasks

Using whole‐head MEG recordings, we were able to evaluate what type of anticipation (perceptual and/or motor) was particularly important for performance in our SRT task. In the literature, there are different proposals regarding exactly what information is acquired – whether more perceptual or motor‐related in nature (Abrahamse *et al*., 2010; Schwarb & Schumacher, 2012). Not surprisingly, the debate has not fully been resolved and, most likely, the nature of the mechanisms will be strongly influenced by specific task parameters and demands. However, it is clear that the sequence of motor responses or learned stimulus‐response rules play an important role in sequence learning (Schwarb & Schumacher, 2012). While it seems that perceptual sequences, in some cases, can be learned independently of an accompanying motor sequence (e.g. Howard *et al*., 1992; Song *et al*., 2008), others failed to show independent perceptual learning (Willingham, 1999).

In the context of the current task, there was surprisingly little evidence of visual anticipation, despite there being ample opportunity for visual spatial orienting given the layout of the stimuli and their correspondence to the finger movements. We found that the utilisation of the learned spatial‐temporal structure proceeded primarily through motor preparation, as was evident in the strong beta modulations that were found over central sites. It may be, however, that the relative contribution of visual and motor areas changes over the course of learning. In the current task, we were not able to assess this possibility as the learning primarily took place in the preceding behavioural session.

To conclude, we have shown that despite the fact that spatial‐temporal predictions remained largely implicit, learned task structure dynamically modulates the time course of lateralised motor cortical beta activity and enhances performance. In future research it will be important to establish how these proactive anticipatory spatial‐temporal modulations emerge and evolve over the course of learning. It will also be important to use even better controlled tasks, to be able to disentangle contributions of different contra‐ and ipsilateral motor areas. In addition, it will be interesting to use more realistic tasks, to see how these predictions hold in more naturalistic settings, where a less arbitrary sequence of behaviours is learned.

## Conflict of interest

The authors declare no competing financial interests.

## Author contributions

SGH and ACN conceived and designed the experiment. SGH acquired the experimental data. SGH and FvE analysed the data. SGH, FvE and ACN interpreted the data, and SGH, FvE and ACN wrote and revised the manuscript.

## Data accessibility

The experimental data will be made available upon request.

**Abbreviations**
EEG: electroencephalography
ERF: event‐related field
MEG: magnetoencephalography
MRI: magnetic resonance imaging
ROI: region‐of‐interest
RSI: response‐to‐stimulus interval
SEM: standard error of the means
SOA: stimulus‐onset asynchrony
SRT: serial reaction time
