# Supplementary material for: Temporal alignment of anticipatory motor cortical beta lateralisation in hidden visual‐motor sequences
Source: Eur J Neurosci. 2017 Nov 6;48(8):2684–95. doi: 10.1111/ejn.13700 (PMC6220967; doi:10.1111/ejn.13700)
Supplement: Supplementary file 1 [file EJN-48-2684-s001.pdf]

EJN European Journal of Neuroscience FENS Federation of European Neuroscience Societies

Temporal alignment of anticipatory motor cortical beta lateralisation in hidden visual-motor sequences

Simone G. Heideman, Freek van Ede & Anna C. Nobre

Review timeline:

Submission date:	03 June 2017
Editorial Decision:	05 July 2017
Revision received:	05 August 2017
Editorial Decision:	22 August 2017
Revision received:	30 August 2017
Accepted:	05 September 2017

Editor: Heleen Slagter

1st Editorial Decision 05 July 2017

Dear Kia,

Your manuscript has been reviewed by external reviewers as well as by the Section Editor, Dr. Heleen Slagter, and ourselves. The reviews collectively indicate that your experiments generated new and important information. However, there are several issues that need to be clarified/resolved before we can consider your manuscript further for publication in EJN.

As you can see, both reviewers recognize that this is an interesting study but both raise a few points that need to be addressed. They both comment on the extreme data reduction undertaken for the MEG analysis. Reviewer 2 also gives some useful suggestions for alternative analyses that would include a larger portion of the data, please address this point carefully.

We also note the following:
- Please remove the figures that are embedded in the text
- Please replace bar charts with more informatice scatter plots or similar (see our recent EJN editorial - Rousselet, Foxe and Bolam - http://onlinelibrary.wiley.com/doi/10.1111/ejn.13400/full)
- Author contributions need to be included in the manuscript itself
- Please re-format the reference list into EJN style

If you are able to respond fully to the points raised, we would be pleased to receive a revision of your paper within 4 weeks.

Thank you for submitting your work to EJN and supporting this Special Issue.

Best wishes

Paul & John
co-Editors in Chief, EJN

Reviews:

Reviewer: 1 (Peter Praamstra, Donders Centre for Cognitive Neuroimaging, The Netherlands)

Comments to the Author
The authors investigate whether the spatial and temporal structure of fixed response sequences, known to result in RT benefits, is also manifested in anticipatory changes of oscillatory brain activity, in particular alpha and beta band activity. The experiment compared behavioural data and oscillatory brain activity, recorded with MEG, between a condition with fixed sequences and a condition with random sequences. The spatial structure of sequences was defined by whether a stimulus and response were left or right. The temporal structure varied in terms of the response-to-stimulus interval (RSI). The behavioural data

confirmed a benefit for fixed vs random sequences, with the benefit being largest for the shortest RSI. The MEG data confirmed anticipatory motor activity in the form of beta power desynchronisation.

This is an interesting study with plausible results. In particular, the MEG effects nicely match the behavioral results. This match is fortunate, since crucial comparisons between repeated sequence and new sequence conditions are based on 118 vs just 20 trials.

Main comments

1. The very unequal number of trials between conditions (per participant and RSI) and the very small number for the new sequence condition are unfortunate, and weaken the results. This weakness of the study may have arisen form the need to carefully match the conditions for response side and RSI on the previous trial (p. 7). Although the imbalance and the small number of trials do not render the study unpublishable, in my view, it needs to be addressed a s a limitation in the discussion.

2. The authors refer to anticipatory oscillatory effects. The changes in beta power in Figure 2c are, however, stronger after than before the button presses. Please comment and explain.

Minor

3. The authors are right that previous work in this area is limited. An early paper by Eimer et al. (1996) was overlooked.

4. Typo p. 13, line 5: ......for occurred.........

Reviewer: 2 (Katja Kornysheva, Bangor University, UK)

Comments to the Author
Review Heidemann, Ede, Nobre 2017, European Journal of Neuroscience

In this MEG study Heidemann et al. evaluate how spatio-temporal expectations during a spatially and temporally structured serial reaction time task (SRTT) modulate beta band dynamics. Specifically the authors show that beta suppression is enhanced for the short vs the long response-stimulus-interval in sensors located above the motor/premotor cortices contralateral to the movement, more so for the repeated than for the new sequences.

Major comments:
This is an interesting and novel study focussing on the dynamic neural control of individual movements in a spatio-temporally structured SRTT. It is a very well written manuscript, thoroughly embedded and discussed within the existing literature.
My major concern is the design and the interconnected data reduction undertaken for the MEG analysis (in contrast to the behavioural analysis), which does not appear convincing in its current form. The authors present data from only two of the three temporal intervals (long/short) leaving out the medium interval, and only from trials where the effector switches from the previous to the current trial (left to right hand; right to left hand) leaving out trials where the effector is repeated. I understood that the rationale behind this approach was to avoid potential confounds in the analysis, as the design introduces differences in the transition probabilities between intervals and hands depending on the trial type (trained/new), p. 7: “conditions differed with regard to the proportion of trials in which the previous response was made with the same or the opposite hand, and differed with regard to the RSI on the previous trial”. This suggests that the new and repeated sequences were structurally different, which may introduce a potential confound of difficulty. Unfortunately, there is no RT baseline measurement for the two trial types (trained; new) that would exclude this argument by showing the same baseline RT levels. Thus learning related RT differences shown between sequences types in the later training phases could be confounded/boosted by structural differences instead of being related to learning-specific effects only. While the data design cannot be tweaked at this stage, the analysis can maximize the data pool by “clamping” the probability of hand switches/repetitions and preceding interval types at the same level for old and new sequences using random trial selection – this would prevent the authors from disregarding such a large proportion of data. In addition, presenting the medium interval would allow them to test whether the stronger beta suppression is a parametric effect and whether its timing can shift (or extend) gradually depending on the interval length. This would be an important addition to the current literature on motor timing and complement the electrophysiological data for sequential tapping in monkeys (e.g. Merchant et al. 2013).

Minor comments:

Methods:

P. 5: Spatial and ordinal is used interchangeably. I would suggest the authors to settle for one of the terms as readers could find this confusing. Spatial seems like a more accurate description, as ordinal could also apply to the order of temporal intervals.

P. 6: “performed independently” instead of “performed independent”

P.7: There is a large difference in the number of trials for the repeated and new sequence conditions (118 vs 20 per RSI), with the new sequence condition naturally more prone to reflect noise components. Given my previous remarks, the analysis could be potentially strengthened by considering trials that have been excluded in the current analysis.

Statistical analysis:

It is not clear from the manuscript why the authors chose to do contrasts instead of a factorial ANOVA – Training (repeated/new) by Interval (Short/Long). Note that the suggested analysis above would include Interval (Short/Medium/Long).

Results:

p. 9: There is no evidence for the statement “participants utilised the learned spatial-temporal sequence structure in the R blocks.” as there is no baseline RT data recorded/presented (see major concern).
Fig. 3: Could the authors please add the topographies from the visual sensor analysis for better comparability with Fig. 2?
Discussion:
P. 13: I do not agree with the authors on that they focussed on utilization whereas previous imaging studies focussed on learning. It might be a semantic issue, but to my understanding previous studies did include what one would call utilization based on sequence knowledge (including the imaging studies by Wymbs et al., Kornysheva et al.). The new contribution here is the focus on utilization of sequence knowledge for the production of individual elements, rather than whole sequences.
P 13: The authors claim that we can make conclusions from the data on how the spatio-temporal information is used by the brain to optimise behaviour, however no data is presented on the correlation between the strength of beta suppression and RT or spatio-temporal accuracy trial-by-trial. Could the authors present such data to substantiate this claim?
P.14: The authors frequently claim that the study examines how temporal and spatial knowledge modulates the neural measures. Meanwhile, the design only allows to make claims with regard to spatio-temporal knowledge, not the main effects of spatial and temporal knowledge separately. This should be made clear in the discussion.

Authors’ Response 05 August 2017

Dear editors, Dear reviewers,

Thank you for having given us the opportunity to revise our manuscript. Based on the useful comments and suggestions of our reviewers, we have made a number of revisions to the manuscript that we believe have helped to raise its quality. For example, following reviewer suggestions, we have added two Figures to our Results (Fig. 4 and 5) that further strengthen our main conclusions. All changes to the manuscript are tracked in blue within the document and are also explicitly pointed out in our replies below. In addition, we now and made sure that our manuscript is in line with the author guidelines of EJN: we have added a graphical abstract, and have included individual data points in all figures that initially only contained bar graphs with standard errors.

We are very grateful for your time and for reconsidering our manuscript.

Yours sincerely,

Simone Heideman, Freek van Ede & Kia Nobre

Reviewer 1

The authors investigate whether the spatial and temporal structure of fixed response sequences, known to result in RT benefits, is also manifested in anticipatory changes of oscillatory brain activity, in particular alpha and beta band activity. The experiment compared behavioural data and oscillatory brain activity, recorded with MEG, between a condition with fixed sequences and a condition with random sequences. The spatial structure of sequences was defined by whether a stimulus and response were left or right. The temporal structure varied in terms of the response-to-stimulus interval (RSI). The behavioural data confirmed a benefit for fixed vs random sequences, with the benefit being largest for the shortest RSI. The MEG data confirmed anticipatory motor activity in the form of beta power desynchronisation.

This is an interesting study with plausible results. In particular, the MEG effects nicely match the behavioral results. This match is fortunate, since crucial comparisons between repeated sequence and new sequence conditions are based on 118 vs just 20 trials.

Main comments

1. The very unequal number of trials between conditions (per participant and RSI) and the very small number for the new sequence condition are unfortunate, and weaken the results. This weakness of the study may have arisen form the need to carefully match the conditions for response side and RSI on the previous trial (p. 7). Although the imbalance and the small number of trials do not render the study unpublishable, in my view, it needs to be addressed a s a limitation in the discussion.

We agree and have added the following to the discussion (p. 14): *"Another limitation of the current task is that due to our trial selection procedure, only a small number of trials (i.e. 20 per RSI) could be included for the new sequence conditions."*

At the same time, we must note that our main contrast of interest involves the contrast between short and long expected RSIs within the repeated condition. For this contrast, we have on average 118 trials per condition. We now also make this more explicit immediately following the added text stated above:

"At the same time, we note that we find an effect despite this limited number, and, moreover, that our main contrast of interest (repeated short vs. long) included 118 trials per RSI."

2. The authors refer to anticipatory oscillatory effects. The changes in beta power in Figure 2c are, however, stronger after than before the button presses. Please comment and explain.

This is indeed the case. As outlined in our introduction, we were particularly interested in anticipatory neural dynamics, and therefore focused on the pre-target (anticipatory) results. However, this does not mean that oscillatory neural activity may not also differ in the post-target/movement period. In fact, given the RT differences, it is perhaps not surprising to see strong modulations also in this window. Critically, however, these modulations already start prior to target onset, in line with our interpretation of an anticipatory modulation by temporal expectations.

Minor

3. The authors are right that previous work in this area is limited. An early paper by Eimer et al. (1996) was overlooked.

Thank you for pointing out this relevant reference. We have added the reference to our discussion (p. 12): *"An EEG study looking at lateralised readiness potentials within learned visual-motor sequences also found that participants showed initial activation of incorrect (but expected) responses when they were presented with a deviant item (Eimer et al., 1996)."*

4. Typo p. 13, line 5: ......for occurred.........

Thank you for pointing out this typo. We changed our manuscript accordingly.

EJN European Journal of Neuroscience FENS Federation of European Neuroscience Societies

Reviewer 2
In this MEG study Heidemann et al. evaluate how spatio-temporal expectations during a spatially and temporally structured serial reaction time task (SRTT) modulate beta band dynamics. Specifically the authors show that beta suppression is enhanced for the short vs the long response-stimulus-interval in sensors located above the motor/premotor cortices contralateral to the movement, more so for the repeated than for the new sequences.

Major comments:
This is an interesting and novel study focussing on the dynamic neural control of individual movements in a spatio-temporally structured SRTT. It is a very well written manuscript, thoroughly embedded and discussed within the existing literature.

My major concern is the design and the interconnected data reduction undertaken for the MEG analysis (in contrast to the behavioural analysis), which does not appear convincing in its current form. The authors present data from only two of the three temporal intervals (long/short) leaving out the medium interval, and only from trials where the effector switches from the previous to the current trial (left to right hand; right to left hand) leaving out trials where the effector is repeated. I understood that the rationale behind this approach was to avoid potential confounds in the analysis, as the design introduces differences in the transition probabilities between intervals and hands depending on the trial type (trained/new), p. 7: “conditions differed with regard to the proportion of trials in which the previous response was made with the same or the opposite hand, and differed with regard to the RSI on the previous trial”. This suggests that the new and repeated sequences were structurally different, which may introduce a potential confound of difficulty. Unfortunately, there is no RT baseline measurement for the two trial types (trained; new) that would exclude this argument by showing the same baseline RT levels. Thus learning related RT differences shown between sequences types in the later training phases could be confounded/boosted by structural differences instead of being related to learning-specific effects only.

We indeed do not show such baseline measurements in this paper, nor do we show that learning of the sequences did indeed take place. In the current manuscript, we focused on the MEG session of a multi-session study. We have previously published a behavioural paper including the behavioural results for all sessions in the study (see Heideman et al., 2017), which demonstrates a clear learning curve for the repeated sequence. The most relevant figures from this previous paper are included below. Session 1 was the behavioural learning session, Session 2 the MEG session, and Session 3 was a MRI session. We copied two relevant Figures from this paper below.

(a)

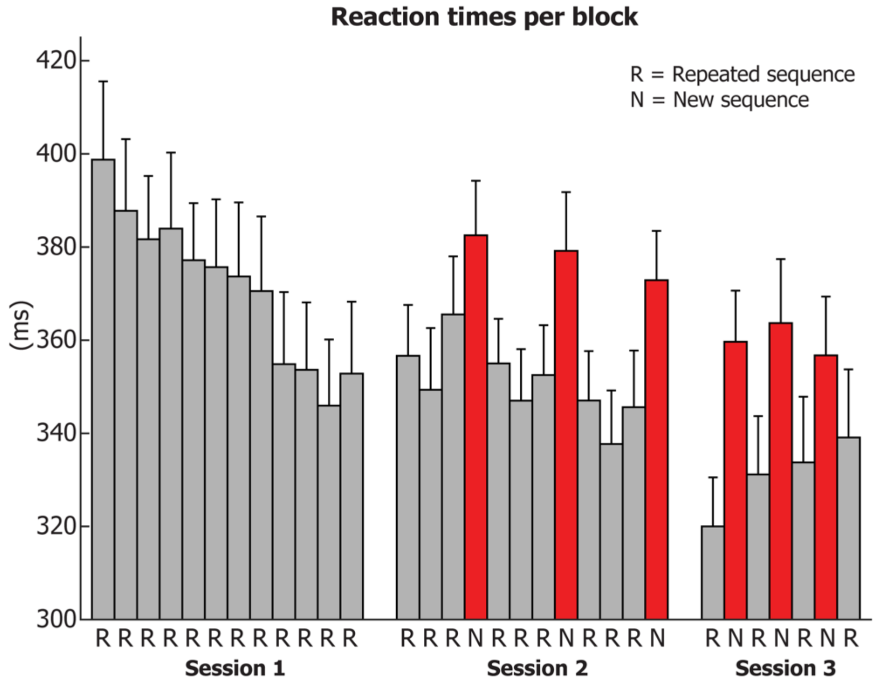

EJN European Journal of Neuroscience FENS Federation of European Neuroscience Societies

Figure 2 *(taken from behavioural paper). Behavioural results. Results are shown for reaction times the first, second and third sessions. Results for blocks with a repeated sequence (R) are shown in grey, while results for blocks where a new sequence (N) was presented are shown in red. Error bars present standard error of means (SEM), calculated using the variance across participants.*

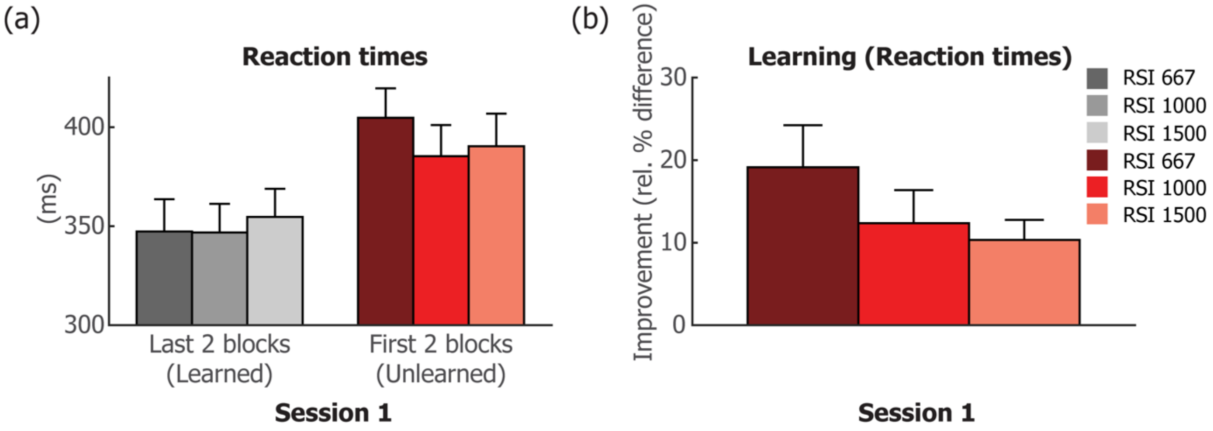

Figure 4 *(taken from behavioural paper). Results are shown for RTs for each of the three response-to-stimulus intervals (RSIs, 667/1000/1500ms), separate for the first two and last two blocks of the first session. The learning effect, shown in (b) was calculated as the relative difference between the first two and the last two blocks, for each RSI length. Error bars present SEM.*

Top plot: Although the new sequence starts out with large RTs (comparable to the RTs in “N” blocks during Session 2 and 3), these decreased strongly (and significantly) over the course of the first behavioural session (that only included repeated blocks), thus suggesting that the enhanced performance in repeated blocks was largely due to the fact that the structure could be learned over the course of the first session.

Bottom plot: This figure shows RTs for the different RSIs during the first two and last two blocks of the behavioural session (which only included “Repeated” blocks). As you can see, the RT improvement over the course of this first session is also largest for the short RSI. At the start of this first session the “Repeated” sequence is still unlearned, and therefore comparable to a “New” sequence, while at the end of this session the sequence has been performed many times and the spatial-temporal structure therefore (implicitly) has been learned. If you compare panel b from this figure with Figure 1d from the current manuscript you can see that the learning effect for the repeated sequence resembles the difference between “Repeated” and “New” in the current data.

These data thus suggest that the main differences in performance between new and repeated blocks were due to the fact that only the repeated blocks contained a *learned* spatial-temporal structure, and not due to structural differences between learned and unlearned sequences per se (indeed, we aimed to match the structural aspects of new and repeated sequences as well as possible; see Methods). Still, we agree that it would have been better to also run a baseline measurement of the blocks that would subsequently be labelled “new” blocks at the start of session 1, as well as to counterbalance (across participants) what sequences would be used as new and repeated sequences. We will take this into account in future studies.

While the data design cannot be tweaked at this stage, the analysis can maximize the data pool by “clamping” the probability of hand switches/repetitions and preceding interval types at the same level for old and new sequences using random trial selection – this would prevent the authors from disregarding such a large proportion of data.

EJN European Journal of Neuroscience FENS Federation of European Neuroscience Societies

We agree that this would be ideal, but unfortunately when adhering to the "hand switch" and "previous medium interval" criteria this is complicated by the fact that there are certain conditions where there are no trials where the previous interval was medium while there was no hand switch.

However, it is critical to point out that our main contrast of interest involves the contrast between short and long expected RSIs, within the repeated condition. For this contrast, we have on average 118 trials per condition. It is only for the contrast between repeated and new trials that we had few trials available (because we only included three new blocks). This analysis may be considered a "bonus", and it was re-assuring to see that a similar difference was observed between the conceptually similar "bonus contrast" between repeated short vs. new short, as between our main contrast of interest between repeated short vs. repeated long.

Also based on a similar concern of reviewer 1, we have now made this concern and clarification explicit in our Discussion (p. 14): *"Another limitation of the current task is that due to our trial selection procedure, only a small number of trials (i.e. 20 per RSI) could be included for the new sequence conditions. At the same time, we note that we find an effect despite this limited number, and, moreover, that our main contrast of interest (repeated short vs. long) included 118 trials per RSI."*

In addition, presenting the medium interval would allow them to test whether the stronger beta suppression is a parametric effect and whether its timing can shift (or extend) gradually depending on the interval length. This would be an important addition to the current literature on motor timing and complement the electrophysiological data for sequential tapping in monkeys (e.g. Merchant et al. 2013).

Thank you for this useful suggestion. We decided to test for parametric effects in our "Repeated" condition, by including a more liberal trial selection (i.e. not using the previous medium criterion). This confirmed a parametric effect, and we have now added this additional results to our manuscript:

(Methods, p. 7):
"[…] *Secondly, to evaluate whether the beta suppression in our experiment might show a parametric effect, we conducted an additional analysis for the repeated sequence condition using all thee RSI lengths. We again only included trials where the previous response was on the opposite hand. For this analysis, it was necessary to relax the criterion that the previous RSI had to be of intermediate length. Contrasts were again calculated as relative contrasts (see above) and evaluated using Fieldtrip's non-parametric cluster-based permutation tests. In addition, we extracted the data of interest in an a-priori defined pre-target window (i.e., beta band activity [15-28 Hz] in the window immediately preceding the first possible target [400 – 667 ms after the previous response]) to directly compare contra vs. ipsi differences between conditions in a repeated-measures ANOVA."*

Results (p. 11):
"*Beta modulations in the medium interval*

To evaluate if beta modulations in our experiment varied parametrically according to the learned RSI, we re-ran our analysis for the repeated condition using all three RSI lengths. Data are shown in Figure 5, for the same motor ROIs that were selected in Figure 2. Figure 5a follows the same convention as Figure 2, except also includes data from the medium RSI condition (note that for this analysis, we had to relax the constraint that the previous RSI should be the medium RSI).

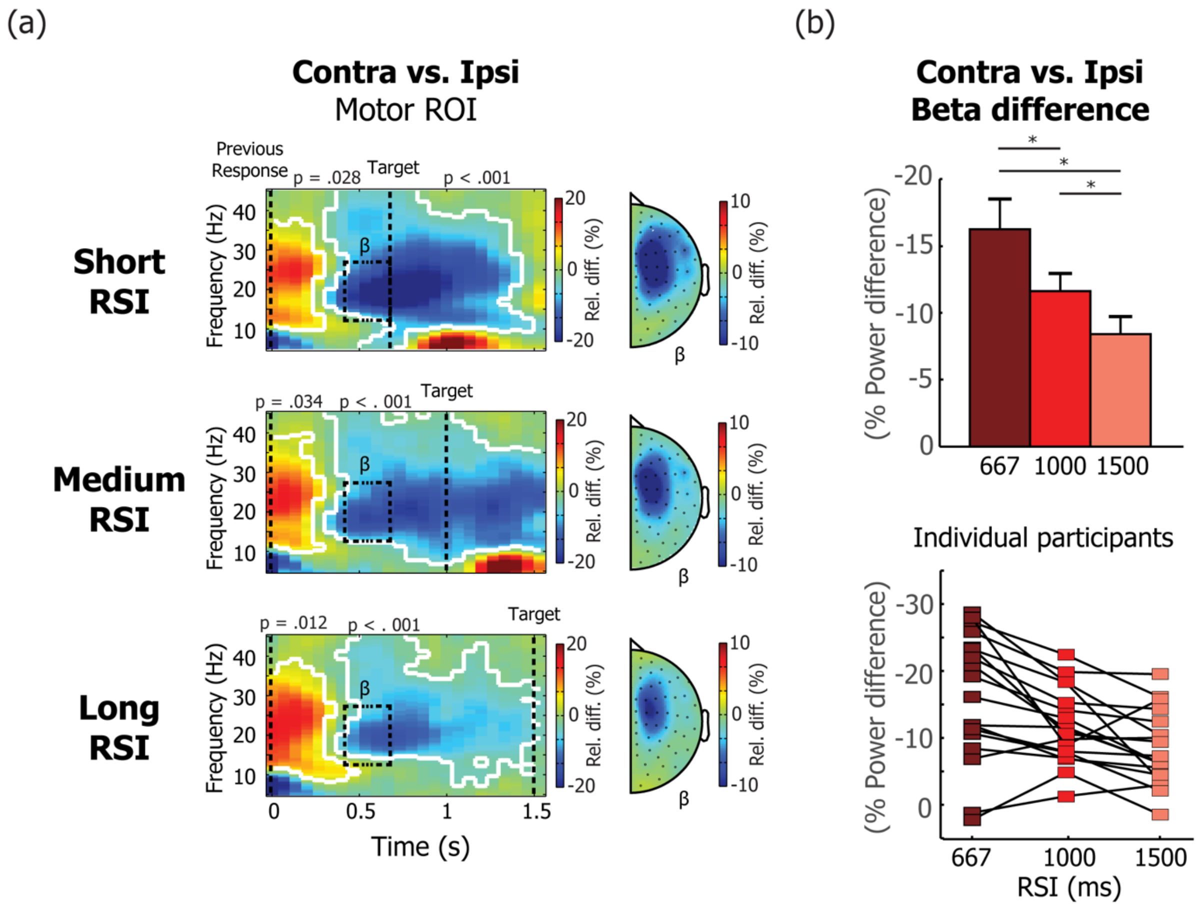

Figure 5. Beta power differences between contralateral and ipsilateral motor channels for all three RSIs. (a) TFR plots reflect frequencies between 5 and 45 Hz, locked to the preceding button press, which always occurred with the opposite hand, compared to the (anticipated) current target. Data are shown for the motor ROI channel selection shown in Figure 2a. Results are shown for targets that occurred either after a short RSI (667 ms; top plot), medium RSI (1000 ms; middle plot) or a long RSI (1500 ms; bottom plot). Significant clusters are outlined in white. Topographies show averages for the beta (15 – 28 Hz) band, for a window between 400 – 667 ms after the button press. (b) Beta power difference between contralateral and ipsilateral motor channels for the same time and frequency selection as used for the topographies in a. The bottom plot shows individual participant data. Asterisks indicate statistically significant effects.

Figure 5b (top) shows the group average of the contra vs. ipsi beta power difference for the same time-frequency window as used for the topographies in Figure 5a, as well as Figure 2. Results for individual participants are shown at the bottom. A repeated-measures ANOVA showed a significant effect of RSI ($F(1,17) = 25.19$, $p < .0001$). Follow-up t-tests showed that the contra vs. ipsi beta power difference (here plotted as a positive effect) was significantly larger for the short, compared to the medium ($t(17) = 3.24$, $p = .005$) and long RSI ($t(17) = 5.02$, $p = .0001$) and also differed significantly between the medium and long RSI ($t(17) = 2.94$, $p = .009$). These results show that the strength of beta modulation differed depending on temporal expectation in a parametric fashion: the earlier a target is expected, the stronger the beta modulation early after the preceding sequence element."

Discussion (p. 11):

"In line with these results, our MEG data revealed anticipatory power modulations in the beta band (lower contra- vs. ipsilateral power) that adapted in a parametric fashion to the expected location and timing of elements within the visual-motor sequence."

(p. 13):

"If you know that a target requiring a response will appear shortly, beta power decreases earlier than when this target is expected only later, similar to the difference between short, medium and long RSIs for the repeated sequence in the current study. Interestingly, interval timing in perceptually guided motor tasks has been reported to be encoded by neurons in medial premotor cortex (e.g., Merchant et al., 2013) which may provide the possible source for instantiating the temporal alignment of the anticipatory beta modulation reported here."

Minor comments:

Methods:
P. 5: Spatial and ordinal is used interchangeably. I would suggest the authors to settle for one of the terms as readers could find this confusing. Spatial seems like a more accurate description, as ordinal could also apply to the order of temporal intervals.

We agree and changed "ordinal" to "spatial" throughout the manuscript.

P. 6: "performed independently" instead of "performed independent"

Thank you. We have corrected this.

P.7: There is a large difference in the number of trials for the repeated and new sequence conditions (118 vs 20 per RSI), with the new sequence condition naturally more prone to reflect noise components. Given my previous remarks, the analysis could be potentially strengthened by considering trials that have been excluded in the current analysis.

We address this concern in response to point 2. In short, our main analysis involves the contrast between short and long expected RSIs within the repeated condition. For this contrast, we did have an average of 118 epochs per condition. We further note that, to our understanding, low trial numbers reduce statistical sensitivity, but do not bias the mean over trials. The observation that the contrast between short repeated vs. new yields a similar effect as the conceptually similar, but statistically more sensitive, contrast between short repeated vs long repeated, suggests to us that the reported result is robust.

Statistical analysis:
It is not clear from the manuscript why the authors chose to do contrasts instead of a factorial ANOVA – Training (repeated/new) by Interval (Short/Long). Note that the suggested analysis above would include Interval (Short/Medium/Long).

Our main analysis involves cluster-based permutation statistics (in order to deal with the multiple comparisons encountered along the temporal and spectral dimensions of our data). As far as we understand (and as far as it appears to be implemented in Fieldtrip, which we used for our analysis), this analysis has been developed for simple contrasts, not ANOVAs. However, that said, the interaction between repeated (short – long) vs. new (short – long) can be reduced to a simple contrast, and we did present and test this interaction effect in figures 2 and 3 (lower right panels). Moreover, as described in response to a comment above, we have now also included an analysis in which we also consider the medium interval (see added Fig. 5) and, for this analysis, we have used an ANOVA to test for parametric effects within an a-priori defined time-frequency cluster.

Results:
p. 9: There is no evidence for the statement "participants utilised the learned spatial-temporal sequence structure in the R blocks." as there is no baseline RT data recorded/presented (see major concern).

We hope that our reply to the first concern alleviates this concern.

Fig. 3: Could the authors please add the topographies from the visual sensor analysis for better comparability with Fig. 2?

Topographies will be the same for both figures; i.e. plots only reflect averages in a selection of either motor or visual channels, whereas topographies show all channels within pre-defined time-frequency windows). For this reason, we included the topographies in Figure 2 only. We anticipate that this was confusing and have

EJN European Journal of Neuroscience FENS Federation of European Neuroscience Societies

therefore added the following sentence to the figure caption of Figure 3: "Corresponding topographies for alpha and beta are shown in Figure 2."

Discussion:
P. 13: I do not agree with the authors on that they focussed on utilization whereas previous imaging studies focussed on learning. It might be a semantic issue, but to my understanding previous studies did include what one would call utilization based on sequence knowledge (including the imaging studies by Wymbs et al., Kornysheva et al.). The new contribution here is the focus on utilization of sequence knowledge for the production of individual elements, rather than whole sequences.

Thank you. We intended this statement to specifically refer to power modulations reported in electrophysiology studies. We agree that this was unclear and changed the sentence to the following (p. 13): *"First, it looks at power modulations reflecting the utilisation of the incidentally learned structure, rather than at the learning per se."*

P 13: The authors claim that we can make conclusions from the data on how the spatio-temporal information is used by the brain to optimise behaviour, however no data is presented on the correlation between the strength of beta suppression and RT or spatio-temporal accuracy trial-by-trial. Could the authors present such data to substantiate this claim?

We thank our reviewer for this very helpful suggestion. We have now calculated this correlation focusing on the short RSIs in our "Repeated" condition (this is the condition where the strongest behavioural effect was found). This analysis indeed suggested that lower beta power in the pre-target interval is associated with faster RTs. We have included this analysis in our manuscript:

Methods (p. 7):
"In addition to the main analysis described above, we performed two post-hoc analyses on our data. First, we evaluated the relation between contra vs. ipsi beta power differences and our behavioural results (RTs) by investigating trial-by-trial Pearson's correlations for the short interval in the repeated condition for each time-frequency point. We limited our analysis to the same subset of trials as described above."

Results (p. 10):
"Trialwise correlations with reaction time

To investigate whether the contra vs. ipsi beta power difference was indeed associated with improved performance, we performed a post-hoc analysis of the correlation between power and RTs, focusing on short RSI trials in repeated blocks. If it is such that lower contra vs. ipsi beta power (putatively reflecting stronger expectation) confers an RT benefit (i.e., lower RT), then this should show as a positive correlation. For this purpose, we calculated the trial-by-trial Pearson correlation for each time-frequency point. Results are shown in Figure 4, with significant clusters outlined in white. These results clearly show a significant correlation both pre- and post-target (two-sided cluster p = .050 pre-target and two-sided cluster p = .020 post target), indicating that participants responded more quickly when there was a larger contra vs. ipsi difference in beta power. In addition, RTs correlated negatively with post-target low-frequency power (putatively, the ERF; p = .038). The topography of this pre-target effect (the effect in which we were particularly interested) again confirmed a localisation to the (pre-)motor areas."

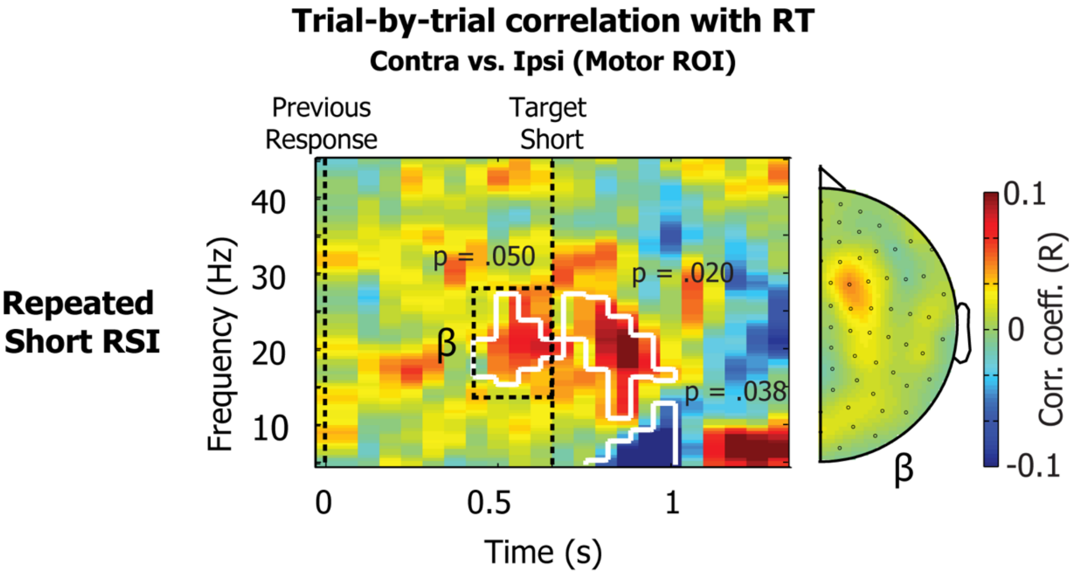

Figure 4. Trial-by-trial correlations between power and RT for the short RSI (667 ms) in the repeated condition. The data show the Pearson correlation coefficient for each time-frequency point between 5 and 45 Hz, for the contra vs. ipsi contrast, locked to the previous button press. Data are plotted for the motor ROI channels shown in Figure 2a. The topography shows the average for the beta (15-28 Hz) frequency band, for a window between 400 – 667 ms after the button press (i.e. just before the presentation of the short target). Significant clusters are outlined in white. The topography reflects results for contralateral vs. ipsilateral activity in symmetrical channel pairs.

Discussion (p. 11):
"We further show that responses to elements that were expected after a short preceding interval were faster in trials in which beta was more suppressed in contralateral (relative to ipsilateral) motor cortical sites – suggesting a role for these anticipatory beta modulations in mediating the observed performance benefits."

P.14: The authors frequently claim that the study examines how temporal and spatial knowledge modulates the neural measures. Meanwhile, the design only allows to make claims with regard to spatio-temporal knowledge, not the main effects of spatial and temporal knowledge separately. This should be made clear in the discussion.

We agree and have added the following to our discussion (P.15): *"Note that the current task does not allow us to make such a distinction, because both spatial and temporal sequences were always present, i.e. we can only establish an effect of spatial-temporal expectations."*

2nd Editorial Decision 22 August 2017

Dear Kia,

Your revised manuscript has been re-evaluated by external reviewers as well as by the Section Editor, Dr. Heleen Slagter and ourselves. We are pleased to inform you that it will be accepted for publication in EJN after a few minor revisions.

As you can see, Reviewer 2 raises a few points that simply need a bit of explanation/clarification which can be relatively easily done. Could you also re-format your graphical abstract into EJN style?

If you are able to respond fully to the points raised, we shall be pleased to receive a revision of your paper within 30 days.

Thank you for submitting your work to EJN and support of this Special Issue.

Best wishes,

Paul & John
co-Editors in Chief, EJN

Reviews:

Reviewer: 2 (Katja Kornysheva, Bangor University, UK)

Comments to the Author
The authors have addressed all my concerns, apart from three:
1. This is a somewhat pedantic point and I am aware that the behavioural paper is already published, but it is still important for the quality of the current manuscript and for those readers who might copy the methods in the future. Improvement in RTs in the trained sequence alone do not constitute clean evidence for sequence-specific learning, or in fact any type of spatio-temporal anticipation through sequence learning. In principle, a large proportion of the improvement would be due to general sequence-unspecific learning effects, such as improvement in visuo-motor transformation – in our own work often 50% of RT improvements. The general gain in RT reduction of

EJN European Journal of Neuroscience FENS Federation of European Neuroscience Societies

trained_beginning vs trained_end in Figure 4 in your response letter is of limited value as the gain likely contains a mixture of both general and sequence-specific effects (the latter leading to the spatio-temporal anticipations of interest). Thus, typically one would compare the improvement in the gain between a trained over a new sequence at the BEGINNING vs the END of training (and any points in between). Since the untrained sequence was only introduced in Session 2, we have to fall back to the MID/END of training gain of trained over new ("probe" costs in your Fig. 1). The leap that we have to make with your study is to trust that the New sequence was very similar in structure and difficulty and that is a clean measure of anticipation due to sequence-specific knowledge. In the previous manuscript you wrote on p. 7: "conditions differed with regard to the proportion of trials in which the previous response was made with the same or the opposite hand, and differed with regard to the RSI on the previous trial". This suggests that the new and repeated sequences were structurally different, which may introduce a potential confound of difficulty on top of anticipation trough sequence knowledge. I would suggest to clarify this point in the manuscript by either arguing against it (if feasible) or specifically highlight that you are not interested in sequence-specific knowledge, but spatio-temporal anticipation in general, regardless of whether the latter is achieved through sequence learning or differences in predictability of the sequence structure.

2. Thank you for providing the parametric analysis in response to my comment. Could you discuss why you tested the parametric effect only for beta amplitude, but not for beta timing (i.e. parametric effect on the onset and duration of beta suppression depending on the interval)?

3. Thank you for providing the very intriguing RT – beta suppression correlation results. You mentioned "significant clusters" – could you outline the cluster correction approach in the corresponding post-hoc Results section (pp 10-11)?

Reviewer: 1 (Peter Praamstra, Donders Centre for Cognitive Neuroimaging, The Netherlands)

Comments to the Author
My comments on the first version of this revised manuscript have been adequately addressed.

Authors' Response 30 August 2017

Dear editors,

Thank you for accepting our manuscript for publication, and for giving us the opportunity to make a final set of minor improvements to the manuscript. All changes to the manuscript are tracked in blue within the document.

The changes we made are as follows:

- First, in reply to point #1. As already mentioned in our previous point-by-point reply, we agree with our reviewer that we ideally would have compared performance on all four sequences at the start of the behavioural session, and additionally, that we should have counterbalanced which sequence would be used as the "repeated" sequence across participants. We also agree that the learning effects likely reflect a mixture of general and sequence specific learning. However, our main contrast of interest, especially for the MEG data, is the short vs. long RSI within the repeated sequence (which involves a learned spatial-temporal expectation). We have now emphasized this again in our Methods section, where we acknowledge the potential differences between our novel and learned sequences: (p.5): "We note that despite our efforts to carefully match the new and repeated sequences, that because each sequence consists of combined spatial and temporal information, there might still be (small) structural or statistical differences between both conditions, based on interference from preceding sequence element-interval combinations. However, this is not a major concern, since our main contrast of interest is the short vs. long RSI within the repeated sequence, which concerns an (implicitly) learned spatial-temporal association. Furthermore, we

EJN European Journal of Neuroscience FENS Federation of European Neuroscience Societies

carefully constrained our analysis, to minimise the influence of the preceding trial (see Time-frequency analysis)."

- In reply to point #2 raised by our reviewer, in our analysis we used the beta amplitude in the early interval as a proxy for the timing of the anticipatory neural dynamics. In other words, if targets are expected early, a larger contra vs. ipsi difference should occur early in the trial, than when the target was only expected later. While the timing of the modulation may have also been quantified directly, we believe that quantifying the magnitude in the early interval will be more reliable, then quantifying the "moment" of modulation (which is not well defined). Moreover, once the early interval passes, temporal expectations can be updated, which may alter the timing and therefore complicate that particular potential analysis. We have now emphasised our magnitude-based approach in our Methods (p.8): "To quantify the influence of temporal expectations, we investigated magnitude (power) differences in the early interval after the previous sequence element, with the rationale that when targets are expected early (as compared to later), power modulations should be more pronounced in the early interval. Therefore, in addition […]",
- As suggested in point #3, we added the cluster correction approach that was used in the post-hoc correlational analysis to the manuscript: (p.7): "These results were evaluated using Fieldtrip's non-parametric cluster-based permutation tests on the correlation values."
- We corrected a typo in one of our ANOVAs (p. 11).
- We removed the heading from our graphical abstract and slightly changed the graphical abstract text to account for the removed information from the figure.

Yours sincerely,

Simone Heideman, Freek van Ede & Kia Nobre
